# hnRNP A1, hnRNP A2B1, and hnRNP K are dysregulated in tauopathies, but do not colocalize with tau pathology

**DOI:** 10.1111/bpa.13305

**Published:** 2024-10-01

**Authors:** Tomas Kavanagh, Kaleah Balcomb, Diba Ahmadi Rastegar, Guinevere F. Lourenco, Thomas Wisniewski, Glenda Halliday, Eleanor Drummond

**Affiliations:** ^1^ Brain and Mind Centre and School of Medical Sciences University of Sydney Camperdown New South Wales Australia; ^2^ Center for Cognitive Neurology and Departments of Neurology, Pathology and Psychiatry Grossman School of Medicine, New York University New York New York USA

**Keywords:** hnRNP, neuropathology, phosphorylated tau, RNA binding proteins, tauopathy

## Abstract

Tau interacts with multiple heterogeneous nuclear ribonucleoproteins (hnRNPs)—a family of RNA binding proteins that regulate multiple known cellular functions, including mRNA splicing, mRNA transport, and translation regulation. We have previously demonstrated particularly significant interactions between phosphorylated tau and three hnRNPs (hnRNP A1, hnRNP A2B1, and hnRNP K). Although multiple hnRNPs have been previously implicated in tauopathies, knowledge of whether these hnRNPs colocalize with tau aggregates or show cellular mislocalization in disease is limited. Here, we performed a neuropathological study examining the colocalization between hnRNP A1, hnRNP A2B1, hnRNP K, and phosphorylated tau in two brain regions (hippocampus and frontal cortex) in six disease groups (Alzheimer's disease, mild cognitive impairment, progressive supranuclear palsy, corticobasal degeneration, Pick's disease, and controls). Contrary to expectations, hnRNP A1, hnRNP A2B1, and hnRNP K did not colocalize with AT8‐immunoreactive phosphorylated tau pathology in any of the tauopathies examined. However, we did observe significant cellular mislocalization of hnRNP A1, hnRNP A2B1 and hnRNP K in tauopathies, with unique patterns of mislocalization observed for each hnRNP. These data point to broad dysregulation of hnRNP A1, A2B1 and K across tauopathies with implications for disease processes and RNA regulation.

## Graphical abstract

Diversity of hnRNP A1, hnRNP A2B1, and hnRNP K immunostaining across hippocampal subregions in Alzheimer's disease.

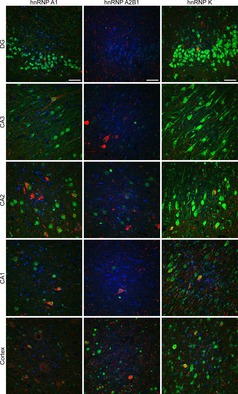

## 1 INTRODUCTION

Tau interactome studies have consistently highlighted a significant interaction between tau and members of the heterogeneous nuclear ribonucleoprotein (hnRNP) family [1]. Specifically, proteomic studies have reported that tau interacts with hnRNP A0, hnRNP A1, hnRNP A2B1, hnRNP A3, hnRNP AB, hnRNP C, hnRNP D, hnRNP DL, hnRNP F, hnRNP H1, hnRNP H2, hnRNP H3, hnRNP K, hnRNP L, hnRNP M, hnRNP R, hnRNP U, hnRNP UL1, and hnRNP UL2 in various tissues including human brain tissue, human cell culture models, rodent tauopathy models, and mouse primary neurons [1, 2, 3, 4, 5, 6, 7, 8, 9]. hnRNPs are a family of RNA binding proteins that have important roles in mRNA transcription, splicing, mRNA stability, translational regulation, and mRNA transport [10]. They have been linked to multiple neurodegenerative diseases including Alzheimer's disease (AD), frontotemporal dementia, and amyotrophic lateral sclerosis (ALS) [11, 12, 13, 14, 15, 16, 17, 18, 19]. While our understanding of the role(s) hnRNPs have in disease pathogenesis is still limited, increasing evidence suggests they have an important role. hnRNPs are typically mislocalized from the nucleus to the cytoplasm in neurons in neurodegenerative disease and aging [11, 15]. Mutations in hnRNPs can cause neurodegenerative disease [18, 20] and hnRNPs can form insoluble aggregates in diseased tissue [21, 22]. hnRNPs can cause splicing changes in disease‐associated proteins or enhance their rate of production to increase the amount of aggregation‐prone tau or APP [23, 24, 25, 26]. ALS‐associated mutations in hnRNP A2B1 expressed in human induced pluipotent stem cells (iPSCs) greatly alter the splicing profiles of those cells [27]. Furthermore, hnRNPs colocalize with and can modify the aggregation of other disease proteins (TAR DNA‐binding protein 43 [TDP‐43], tau) [5, 6, 15, 17, 28].

We were particularly interested to study the interaction between tau and three specific hnRNPs (hnRNP A1, hnRNP A2B1 and hnRNP K), as we previously found these to significantly interact with disease‐associated phosphorylated tau (pTau) in human AD brain tissue [2]. In addition, previous bulk tissue proteomic studies have reported that these three hnRNPs are significantly increased in AD in the frontal cortex [29, 30, 31, 32]. We hypothesized that the interaction between pTau and hnRNP A1, hnRNP A2B1, and hnRNP K had an important role in AD pathogenesis and could play a role in tau aggregation. This was, in part, based on our previous study showing that other novel pTau interactors identified in our proteomics study strongly colocalize with tau aggregates in AD [33] and further supported by a recent study that showed evidence of increased complexes of hnRNP A2B1‐oligomeric tau in human brain tissue at early stages of AD [5].

Previous studies examining the colocalization between tau aggregates and hnRNPs in human brain tissue are limited. One study showed that hnRNP K did not colocalize with pTau aggregates in the frontal cortex from cases with frontotemporal lobar degeneration with tau pathology (FTLD‐Tau) [11], whereas another showed that hnRNP A2B1 colocalized with oligomeric tau in the temporal cortex of AD patients, primarily at Braak stages II–IV [5]. Dysregulation of these proteins can have far‐reaching implications because of their role in regulating mRNA metabolism. Therefore, this study aimed to determine if hnRNP A1, hnRNP A2B1, and hnRNP K colocalized with tau aggregates in human brain tissue in a range of tauopathies including AD, mild cognitive impairment (MCI), corticobasal degeneration (CBD), Pick's disease (PiD), and progressive supranuclear palsy (PSP).

## 2 METHODS

### 2.1 Human brain tissue samples

All cases used in this study were obtained from the New York University Alzheimer's Disease Center, USA, and the Sydney Brain Bank, Australia, both of which supply human brain tissue from ethically approved longitudinally assessed regional brain donor programs on neurodegenerative diseases. All procedures were performed under protocols approved by the New York University Alzheimer Disease Center, NY, the Southeastern Sydney and Illawarra Local Health District and the Universities of New South Wales and Sydney, Australia. In all cases, written informed consent for research was obtained from the patient or legal guardian, and the material used had appropriate ethical approval for use in this project. All patients' data and samples were coded and handled according to National Institutes of Health (USA) and National Health and Medical Research Council (Australia) guidelines to protect patients' identities. All case‐specific patient data is included in Table 1.

**TABLE 1:** Patient data.

Case ID	ABC score	Age	Sex	Region	hnRNP A1	hnRNP A2/B1	hnRNP K
AD #1	A3, B3, C3	82	F	Hippocampus	Yes	Yes	Yes
AD #2	A3, B3, C3	85	F	Hippocampus	Yes	Yes	Yes
AD #3	A3, B3, C3	72	F	Hippocampus	Yes	Yes	Yes
AD #4	A3, B3, C3	73	M	Hippocampus	Yes	Yes	Yes
AD #5	A3, B3, C3	89	F	Hippocampus	Yes	Yes	Yes
AD #6	A3, B3, C3	92	F	Hippocampus	Yes	Yes	Yes
MCI #1	A1, B2, C1	86	F	Hippocampus	Yes	Yes	Yes
MCI #2	A1, B2, C1	85	F	Hippocampus	Yes	Yes	Yes
MCI #3	A2, B1, C1	66	M	Hippocampus		Yes	
MCI #4	A1, B1, C1	90	M	Hippocampus			Yes
CBD #1	A1, B2, C0	87	F	Frontal cortex	Yes	Yes	Yes
CBD #2	A0, B2, C0	53	M	Frontal cortex	Yes	Yes	Yes
CBD #3	A0, B1, C0	73	M	Frontal cortex	Yes	Yes	Yes
CBD #4	A1, B1, C0	76	F	Frontal cortex		Yes	Yes
CBD #5	A0, B2, C0	73	M	Frontal cortex	Yes	Yes	Yes
PiD #1	A3, B0, C0	82	F	Frontal cortex	Yes	Yes	Yes
PiD #2	A0, B0, C0	74	M	Frontal cortex	Yes	Yes	Yes
PiD #3	A0, B1, C0	66	F	Frontal cortex	Yes	Yes	Yes
PiD #4	A0, B0, C0	73	M	Frontal cortex	Yes	Yes	Yes
PiD #5	A0, B2, C0	82	M	Frontal cortex	Yes	Yes	Yes
PiD #6	A0, B2, C0	65	F	Hippocampus	Yes	Yes	Yes
PiD #7	A0, B0, C0	69	M	Hippocampus	Yes	Yes	Yes
PiD #8	A0, B0, C0	71	F	Hippocampus		Yes	Yes
PiD #9	A0, B0, C0	67	M	Hippocampus		Yes	Yes
PSP #1	A1, B0, C0	76	F	Frontal cortex	Yes	Yes	Yes
PSP #2	A1, B1, C0	70	M	Frontal cortex	Yes	Yes	Yes
PSP #3	A2, B1, C0	76	M	Frontal cortex	Yes	Yes	Yes
PSP #4	A0, B1, C0	74	F	Frontal cortex	Yes	Yes	Yes
PSP #5	A2, B1, C0	70	F	Frontal cortex	Yes	Yes	Yes
Control #1	A0, B1, C0	79	M	Frontal cortex	Yes	Yes	Yes
Control #2	A2, B1, C3	88	F	Frontal cortex	Yes	Yes	Yes
Control #3	A2, B1, C1	84	M	Frontal cortex	Yes	Yes	Yes
Control #4	A1, B0, C0	84	M	Frontal cortex	Yes	Yes	Yes
Control #5	A1, B1, C0	80	F	Frontal cortex	Yes	Yes	Yes
Control #6	A0, B0, C0	77	M	Hippocampus	Yes	Yes	Yes
Control #7	A1, B0, C0	59	M	Hippocampus	Yes	Yes	Yes
Control #8	n/a	71	F	Hippocampus	Yes	Yes	Yes
Control #9	A1, B1, C1	89	M	Hippocampus	Yes	Yes	Yes
Control #10	A0, B1, C0	76	M	Hippocampus		Yes	
Control #11	A0, B1, C0	88	M	Hippocampus	Yes		

*Note*: Heterogeneous nuclear ribonucleoprotein (hnRNP) columns indicate cases that were costained with phosphorylated tau (pTau) (AT8) and included in each analysis.

Abbreviations: AD, Alzheimer's disease; CBD, corticobasal degeneration; F, female; MCI, mild cognitive impairment; PiD, Pick's disease; M, male; PSP, progressive supranuclear palsy; n/a, not available.

### 2.2 Fluorescent immunohistochemistry

Fluorescent immunohistochemistry was performed on formalin‐fixed paraffin‐embedded tissue sections as described previously [33, 34]. Briefly, 8 μm sections were deparaffinized and rehydrated by a series of xylene and ethanol washes. Antigen retrieval was performed by boiling in citrate buffer (10 mM sodium citrate, 0.05% Tween‐20; pH 6) in a microwave for 21 min. Sections were blocked with 10% normal goat serum and incubated overnight at 4°C with hnRNP A2B1 (1:150; Invitrogen; catalog #PA5‐88180), hnRNP A1 (1:75; Abcam; catalog # ab232824), and hnRNP K (1:150; Invitrogen; catalog #PA5‐95866) primary antibodies in combination with the anti‐pTau antibody AT8 (1:500; Thermo Fisher Scientific; catalog #MN1020). Sections were then incubated for 2 h at room temperature with anti‐rabbit‐488 (1:500, Jackson ImmunoResearch) and anti‐mouse‐647 (1:500, Jackson ImmunoResearch), validation studies used anti‐chicken‐488 (1:500, Invitrogen), anti‐goat‐647 (1:1000, Invitrogen), anti‐Rabbit‐488 (1:1000, Sigma), and anti‐Rabbit‐647 (1:500, Jackson ImmunoResearch) where appropriate. All slides were counterstained with Hoechst 33342 (0.01 mg/mL; Sigma; catalog #B2261) to label nuclei and coverslipped (ProLong™ Diamond Antifade Mountant, Invitrogen). Immunohistochemistry was performed in three batches per staining combination and each batch included a no primary antibody control which were only treated with secondary antibodies (Figure S1). Serial sections of a positive control sample (AD #1) were included in all batches to identify any batch inconsistencies. Cases from each experimental group were evenly distributed into each staining batch. For follow‐up staining of hnRNPs with other tau epitopes, *n* = 1 slide from AD and MCI in the hippocampus and *n* = 1 CBD, PiD, and PSP were used from the frontal cortex. Slides were stained for hnRNPs using the method described above in combination with MC1 (1:100, misfolded tau, a gift from Peter Davies), AT270 (pT181, 1:500; Thermo Fisher, catalog #MN1050) or AT180 (pT231, 1:500; Thermo Fisher, catalog #MN1040) primary antibodies. To determine cell type expression of hnRNP K and hnRNP A2B1, hippocampus or frontal cortex sections, respectively, were stained for *n* = 1 AD and *n* = 1 CBD case (as these were the most prolific cases of possible non‐neuronal cell expression of hnRNPs). These slides were costained for hnRNP K (AD, hippocampus) and hnRNP A2B1 (CBD, frontal cortex) and glial fibrillary acidic protein (GFAP, 1:1000, Novus Biologicals, catalog #NOVNBP105198), ionized calcium binding adaptor molecule 1 (IBA1, 1:250, Abcam, catalog #ab5076), or oligodendrocyte transcription factor 2 (OLIG2, 1:250, R&D Systems, catalog #AF2418) primary antibodies. Validation and cell type studies were coverslipped with ProLong™ Glass Antifade Mountant (Invitrogen).

### 2.3 Imaging and analysis

Sections were imaged on a Nikon C2 confocal microscope at 40x magnification (0.95 NA) with z‐stacks set to 1.5 μM to sample the tissue thickness (8 μM). Five images of frontal cortex sections were acquired targeting cortical layers II‐III throughout the section. The following images were acquired for each hippocampal section: two images of the dentate gyrus (DG), CA2, CA1, and subiculum (Sb), one image each of CA4 and CA3, and five images of layers II–III throughout the entorhinal and temporal cortices (Cx) present at this level of the hippocampus (Figure S1).

The TRITC channel was imaged with the same settings as FITC to profile autofluorescence. Autofluorescence was removed from all other channels by subtracting the TRITC channel. Background noise was then removed with the ‘rolling ball’ algorithm and final cleaned images were saved for further analysis. Images were processed in ImageJ to select the brightest z plane in the FITC channel (hnRNP stain), which were then used for image quantification analysis. ImageJ was used to calculate integrated density, count of particles and size of particles for both hnRNPs and AT8 using the measure and summarize particle functions. For hnRNPs, this was performed on the brightest stack. pTau (AT8) percentage load was calculated based on percentage area with signal above a manually defined threshold of the maximum projection of all stacks in a converted 8‐bit image (threshold of 40 for frontal cortex, 100 for hippocampus). Colocalization analysis was performed on the full 16‐bit depth images after autofluorescence and noise removal. Colocalization (Pearson's method) was assessed with the ‘coloc2’ plugin within a mask defined by pTau positive areas (threshold set to 40). Colocalization *p*‐values were computed with Coste's method of randomization with 100 rounds. To determine the percentage of neurons positive for hnRNPs, frames were manually counted. The CA2 subregion of the hippocampus and all images of the frontal cortex were manually counted for positive neuronal and glial signal and binned into nuclear and/or cytoplasmic signal. In this analysis, cells were selected based on nuclear morphology: large and complex nuclei were denoted as neurons, whereas small and simple nuclei were designated ‘glia’. The total number of neurons and glia was counted, as well as the number of neurons/glia with nuclear signal and the number with cytoplasmic signal. The CA2 region was selected for manual counting as it had two images acquired per sample, discernible cells with minimal overlap, and was not affected by extreme cell loss.

For compartment colocalization studies, slides were first scanned on the Olympus VS‐200 slide scanner at ×40 magnification. Representative confocal images of the DG, CA4, CA3, and CA2 were then taken with ×60 objective (numerical aperture [NA] 1.40) with ×2.5 digital zoom (maximum pixel size of 0.1 μM) and 0.1 μM z‐stack heights. The TRITC channel was imaged to profile and subtract autofluorescence from all other channels. For cell type expression analysis, slides were first scanned on the Olympus VS‐200 slide scanner at ×40 magnification. Representative confocal images of marked cells were taken with a ×60 objective (NA 1.40), and 0.5 μM z‐stack heights to capture localization of hnRNPs in cell processes. For alternative tau epitope colocalization imaging studies, slides were first scanned on the Olympus VS‐200 slide scanner at ×40 magnification. Representative confocal images of the DG, CA4, CA3, and CA2 were then taken with ×60 objective (NA 1.40) with 1.5 μM z‐stack heights to sample tau puncta throughout the section.

### 2.4 Statistics and graphing

Data processing, statistics, and graphing were performed in R using the tidyverse v2.0.0 package, RColorBrewer v 1.1–3, ggpubr v0.6.0, and FAS v0.9.5. The covariates of age, sex, and postmortem delay were checked for any significant imbalances between disease groups (Figure S2). As groups were unbalanced and not normally distributed (Shapiro–Wilk test), we used the Kruskal–Wallis test to assess for group‐wide differences in positive nuclear percentages followed by Dunn's test to determine the significance of multiple comparisons. Graphs were edited in Adobe Illustrator v28.1. Micrograph panels were constructed in Adobe Photoshop v25.4 and brightness increased uniformly for green and red channels for presentation purposes.

## 3 RESULTS

### 3.1 hnRNP A1, hnRNP A2B1, and hnRNP K do not colocalize with large AT8‐immunoreactive phosphorylated tau aggregates

**FIGURE 1:**
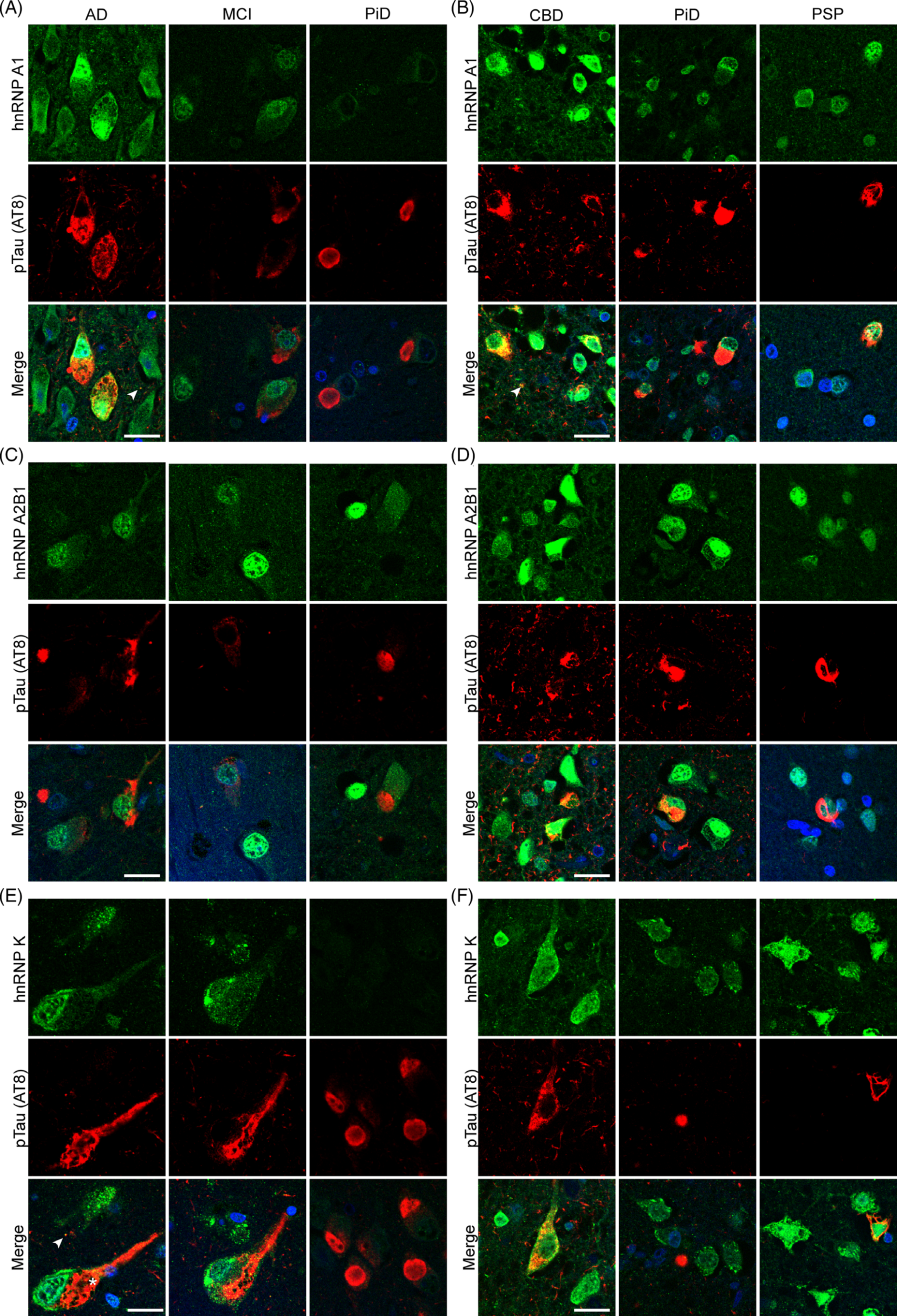
Colocalization of phosphorylated tau (pTau) and heterogeneous nuclear ribonucleoproteins (hnRNPs). (A) Colocalization of hnRNP A1 and pTau in Alzheimer's disease (AD), mild cognitive impairment (MCI), and Pick's disease (PiD) in the hippocampus. (B) Colocalization of hnRNP A1 and pTau in corticobasal degeneration (CBD), PiD, and progressive supranuclear palsy (PSP) in the frontal cortex. (C) Colocalization of hnRNP A2B1 and pTau in AD, MCI, and PiD in the hippocampus. (D) Colocalization of hnRNP A2B1 and pTau in CBD, PiD, and PSP in the frontal cortex. (E) Colocalization of hnRNP K and pTau in AD, MCI, and PiD in the hippocampus. (F) Colocalization of hnRNP K and pTau in CBD, PiD, and PSP in the frontal cortex. Controls are not shown as they have no tau pathology in the selected regions. Scale bar = 20 μM. Red = pTau (immunostained with AT8), Green = hnRNP. Arrowheads point to colocalization between small pTau puncta and hnRNPs. *Example of large neurofibrillary tangle with no hnRNP colocalization and exclusionary staining patterns. Images are the brightest slice.

To determine if hnRNP A1, hnRNP A2B1, or hnRNP K colocalize with pTau aggregates in tauopathies, we costained human postmortem tissue sections for phosphorylated tau (AT8 antibody; pTau S202/T205) and each of the target hnRNPs. Immunohistochemistry was performed in brain regions containing abundant tau pathology in each disease; hippocampus for AD, MCI, and PiD and frontal cortex for CBD, PSP, and PiD. Sections of age‐matched cognitively normal tissue from both the hippocampus and frontal cortex were included as control cases. Fifteen confocal images from seven regions were acquired from each hippocampal section (2× DG, 1× CA4, 1× CA3, 2× CA2, 2× CA1, 2× subiculum and 5× cortical regions). Five images were acquired from frontal cortex sections from different locations throughout the section in cortical layers II–III. We observed no significant colocalization between any hnRNP tested and pTau (Figure 1 and Figure S3). While strong cytoplasmic hnRNP staining was often observed in cells containing pTau tangles, hnRNP staining often appeared mutually exclusive of pTau staining, with the strongest hnRNP staining being observed in intracellular regions not immunoreactive for pTau (for example Figure 1E). This finding was consistent in all tauopathies tested and observed in both hippocampus and frontal cortex. While there was infrequent colocalization between small pTau aggregates and hnRNPs in the cytoplasm, this appeared to be coincidental, rather than direct colocalization (Figure 1, arrows, Figure S3). There was no colocalization between hnRNPs and pTau in dystrophic neurites (Figure 1), although there does appear to be infrequent colocalization with hnRNPs (mostly hnRNP A2B1 and hnRNP K) in neuropil threads and small pTau puncta, predominantly in AD (Figures 3A, 4A, and 5A). This lack of hnRNP‐tau colocalization was despite the presence of abundant, disease‐specific tau pathology in all tauopathy cases. pTau pathology was individually quantified in each subregion (DG, CA4, CA3, CA2, CA1, subiculum, temporal cortex, frontal cortex) in each of the three serial sections from each case that were costained with hnRNP A1 (Figure 3D,E), hnRNP A2B1 (Figure 4D,E) and hnRNP K (Figure 5D,E). pTau staining was highly consistent between each of the staining runs, with the expected regional differences in levels of pTau staining for each disease: MCI cases showed highest pTau staining in the subiculum, followed by CA1, CA2 and temporal cortex; AD cases showed moderate–high pTau staining in all hippocampal regions studied, with highest levels being observed in the subiculum, CA2 and temporal cortex; and PiD showed highest pTau staining in the DG, CA1, subiculum, and temporal cortex. In frontal cortex sections, CBD and PiD cases showed the highest levels of pTau pathology, with comparatively lower levels being observed in PSP cases.

### 3.2 hnRNPs are dysregulated in neurodegenerative disease

**FIGURE 2:**
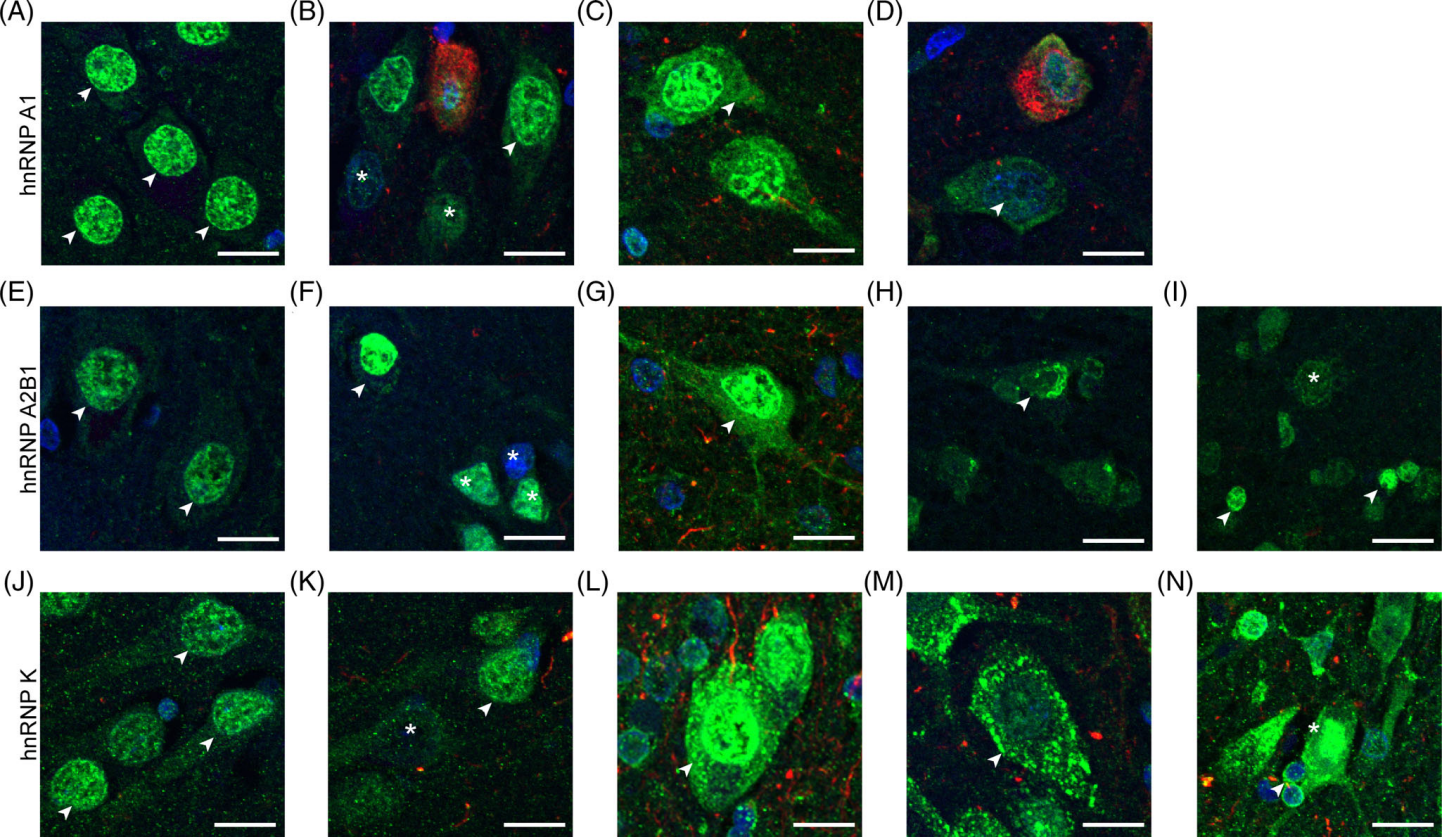
Examples of dysregulation of heterogeneous nuclear ribonucleoproteins (hnRNPs) across disease states. Cropped micrographs giving examples of hnRNP dysregulation in example cells present in either the hippocampus or frontal cortex; (A) hippocampal cells showing normal nuclear expression of hnRNP A1 (arrows). (B) Hippocampal cells showing altered expression levels of hnRNP A1 (arrows = high expression, * = low expression). (C) Hippocampal cells showing cytoplasmic mislocalization of hnRNP A1 (arrow). (D) Hippocampal cell showing nuclear depletion of hnRNP A1 with cytoplasmic mislocalization. (E) Hippocampal cells showing normal nuclear expression of hnRNP A2B1 (arrows). (F) Frontal cortex cells showing altered expression levels of hnRNP A2B1 (arrows = high expression, * = low expression). (G) Hippocampal cell showing cytoplasmic mislocalization of hnRNP A2B1 (arrow). (H) Frontal cortex cell showing nuclear depletion of hnRNP A2B1 with cytoplasmic puncta (arrow). (I) Altered cell type expression profile (glial cells) of hnRNP A2B1 in the frontal cortex in disease (glial cells arrows, * = neighboring neuron). (J) Hippocampal cells showing normal nuclear expression of hnRNP K (arrows). (K) Hippocampal cells showing altered expression levels of hnRNP K (arrows = high expression, * = low expression). (L) Hippocampal cell showing cytoplasmic mislocalization of hnRNP K with cytoplasmic puncta (arrow). (M) Hippocampal cell showing nuclear depletion of hnRNP K with cytoplasmic puncta (arrow). (N) Hippocampal cells showing altered cell type expression profile (glial cells arrows, * = neighboring neuron) of hnRNP K in disease. Scale bar = 15 μM, blue = nuclei (Hoescht), green = hnRNP, Red = phosphorylated tau (pTau) (immunostained with AT8). Images are cropped maximum projections.

Despite the lack of colocalization with pTau, mislocalization of hnRNP A1, hnRNP A2B1, and hnRNP K was a prominent feature of all tauopathies examined. hnRNP mislocalization was characterized by complex morphological changes that were observed on a cell‐by‐cell basis. Often all hnRNP mislocalization profiles were observed in each case, meaning that no consistent mislocalization phenotype could be defined for each tauopathy. hnRNP A1 staining profiles ranged from cells with strong nuclear staining (Figure 2A), cells with minimal/no staining (Figure 2B), cytoplasmic mislocalization (Figure 2C), and nuclear depletion with cytoplasmic mislocalization (Figure 2D). hnRNP A2B1 staining profiles ranged from normal nuclear expression (Figure 2E), cells with minimal/no staining (Figure 2F), cytoplasmic mislocalization (Figure 2G), nuclear depletion with rare cytoplasmic puncta (Figure 2H) and expression in atypical cell populations in disease (Figure 2I). Based on their small size, these atypical cells were hypothesized to be glia and were commonly observed in the frontal cortex rather than the hippocampus. hnRNP K staining profiles ranged from normal nuclear expression (Figure 2J), cells with minimal/no staining (Figure 2K), punctate cytoplasmic mislocalization (Figure 2L), nuclear depletion with cytoplasmic puncta (Figure 2M), as well as expression in atypical cells in disease states, mostly peri‐nuclear expression in glial cells (Figure 2N). Of these staining profiles, the strong nuclear staining profile (considered to be the physiological profile for hnRNP staining) was commonly observed for hnRNP A1 and hnRNP A2B1 across all disease groups. This strong nuclear staining profile was less frequently observed for hnRNP K, even in control cases, suggesting widespread mislocalization of hnRNP K in our cohort, irrespective of disease, and may therefore be a factor of aging.

To further investigate the atypical cell expression patterns observed for hnRNP A2B1 (Figure 2I) and hnRNP K (Figure 2N), we stained select cases with markers for astrocytes (GFAP), microglia (IBA1) and oligodendrocytes (OLIG2). hnRNP A2B1 was generally not observed in GFAP‐positive astrocytes or IBA1‐positive microglia in the frontal cortex examples (Figure S4a–c). However, OLIG2‐positive oligodendrocytes were faintly positive for hnRNP A2B1 (Figure S4c). However, there remained a population of cells that showed strong hnRNP A2B1 staining but were not positive for the three cell type markers used here. In the AD hippocampus (the most prevalent cases with possible non‐neuronal cell expression of hnRNP K), we observed very minimal hnRNP K expression in GFAP‐positive astrocytes (Figure S4d). However, we observed hnRNP K in both IBA1‐positive microglia and OLIG2‐positive oligodendrocyte cells (Figure S4e,f). The IBA1‐positive microglia displayed potential phagocytic activity of hnRNP K puncta, with hnRNP K puncta observable throughout the cell body and at the end of cell processes (Figure S4e). The OLIG2‐positive cells displayed predominantly perinuclear expression of hnRNP K but there remained a population of small nucleus, hnRNP K‐positive cells that were not positive for any of the cell markers assessed (Figure S4d–f).

**FIGURE 3:**
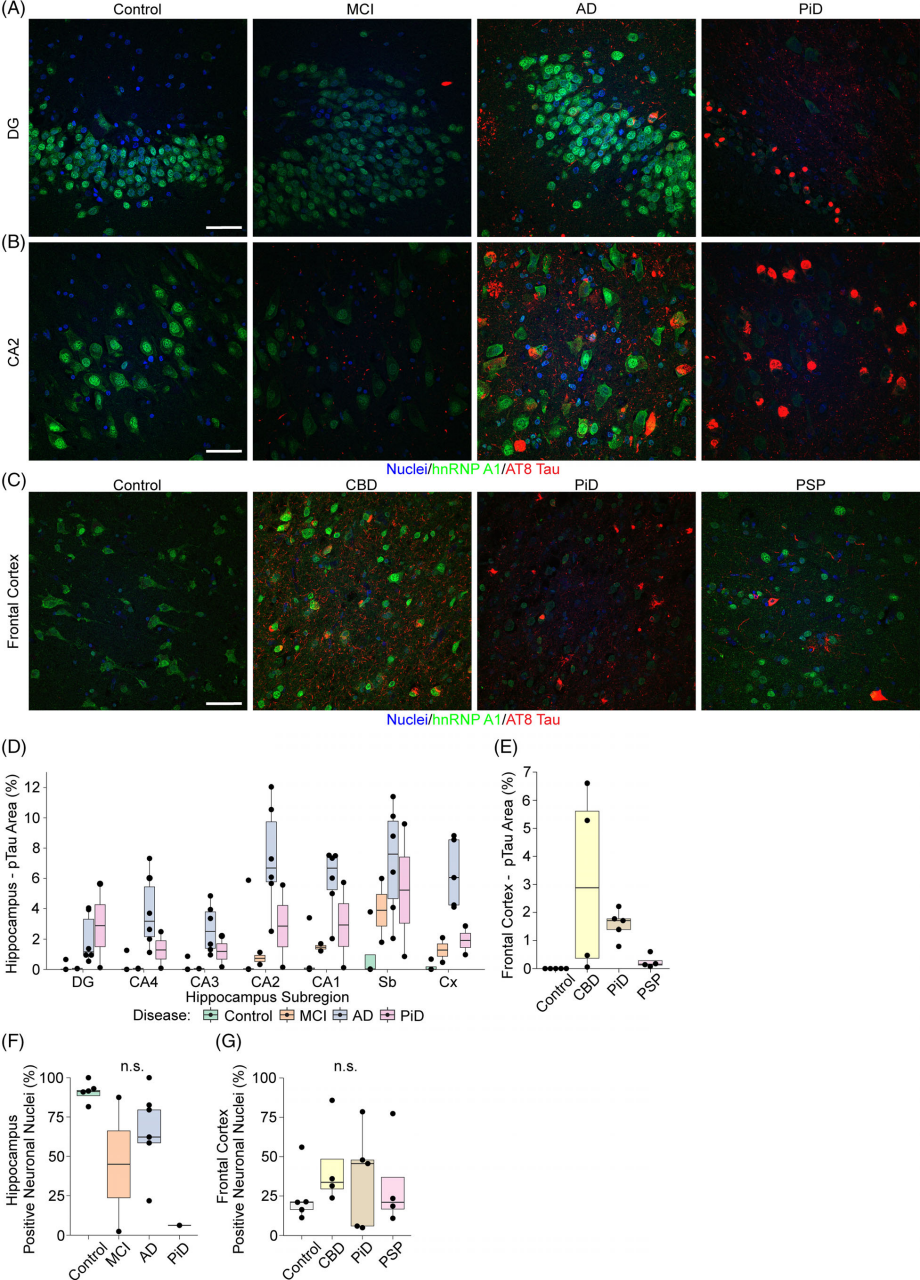
Heterogeneous nuclear ribonucleoprotein (hnRNP) A1 expression patterns. Composite micrographs of (A) the dentate gyrus (DG) in the hippocampus from control, mild cognitive impairment (MCI), Alzheimer's disease (AD), and Pick's disease (PiD) cases stained for hnRNP A1, phosphorylated tau (pTau) (AT8), and Hoescht (nuclei); (B) micrographs of the CA2 subregion of the hippocampus from control, MCI, AD, and PiD cases stained for hnRNP A1, pTau (AT8), and Hoescht (nuclei); (C) micrographs of the frontal cortex from control, corticobasal degeneration (CBD), PiD, and progressive supranuclear palsy (PSP) cases stained for hnRNP A1, pTau (AT8), and Hoescht (nuclei). (D) Boxplots showing the percentage load (percentage area with positive staining) of pTau across hippocampal subregions for controls (*n* = 4), MCI (*n* = 2), AD (*n* = 6), and PiD (*n* = 2). (E) Boxplots showing the percentage load (area) of pTau in the frontal cortex of Controls (*n* = 5), CBD (*n* = 4), PiD (*n* = 5), and PSP (*n* = 4). (F) Boxplots showing the percentage of neurons with positive nuclear staining of hnRNP A1 in each group (counted manually from the CA2 subregion of the hippocampus, controls *n* = 4, MCI *n* = 2, AD *n* = 6 and PiD *n* = 1). (G) boxplots showing the percentage of neurons with positive nuclear stain for hnRNP A1 in the frontal cortex for each group (Controls *n* = 5, CBD *n* = 4, PiD *n* = 5 and PSP *n* = 4). Scale bar = 50 μM. All images are maximum projections. Boxplots represent the median values, lower and upper quartiles (75% and 25%, respectively) and whiskers represent the quartile to minimum/maximum value but extending no further than 1.5 times the interquartile range. Abbreviations: CA, cornu ammonis; n.s., not significant; Sb, subiculum; DG, dentate gyrus; Cx, cortex

In the hippocampus, hnRNP A1 expression was prominent in the dentate gyrus (DG), with most cells showing strong positive staining for hnRNP A1 (Figure 3A). While hnRNP A1 staining intensity was high in the DG and CA1‐4 in control and AD cases, hnRNP A1 levels trended lower in MCI and were visually depleted in PiD in the DG (Figure 3A). The proportion of pyramidal cells displaying hnRNP A1 staining in CA1‐4 appeared to be affected by disease. For example, nearly all CA2 pyramidal cells in control cases displayed hnRNP A1 staining with a profile of strong nuclear expression and mild cytoplasmic staining (Figure 3B). In comparison, hnRNP A1 levels were variably altered in MCI, AD, and PiD, ranging from increased cytoplasmic levels with or without nuclear depletion in AD to widespread depletion throughout the cell in PiD. hnRNP A1 staining in the frontal cortex was less prominent than in the hippocampus (Figure 3C). In controls, most frontal cortex neurons had primarily cytoplasmic expression of hnRNP A1, whereas CBD and PSP had the most prevalent nuclear expression, albeit with a large degree of cytoplasmic mislocalization (Figure 3C). The total number of hnRNP A1‐positive neuronal nuclei was manually counted in CA2 and frontal cortex images. While no significant differences in the percentage of hnRNP A1 positive neuronal nuclei in either CA2 or frontal cortex were observed (Figure 3F,G), CA2 in controls had near ~90% positivity rate compared to variable rates in other diseases that were decreased in most cases. Interestingly the number of hnRNP A1‐positive neuronal nuclei in controls was much lower in the frontal cortex in comparison to the hippocampus, with only ~20% of neurons showing nuclear hnRNP A1 staining.

**FIGURE 4:**
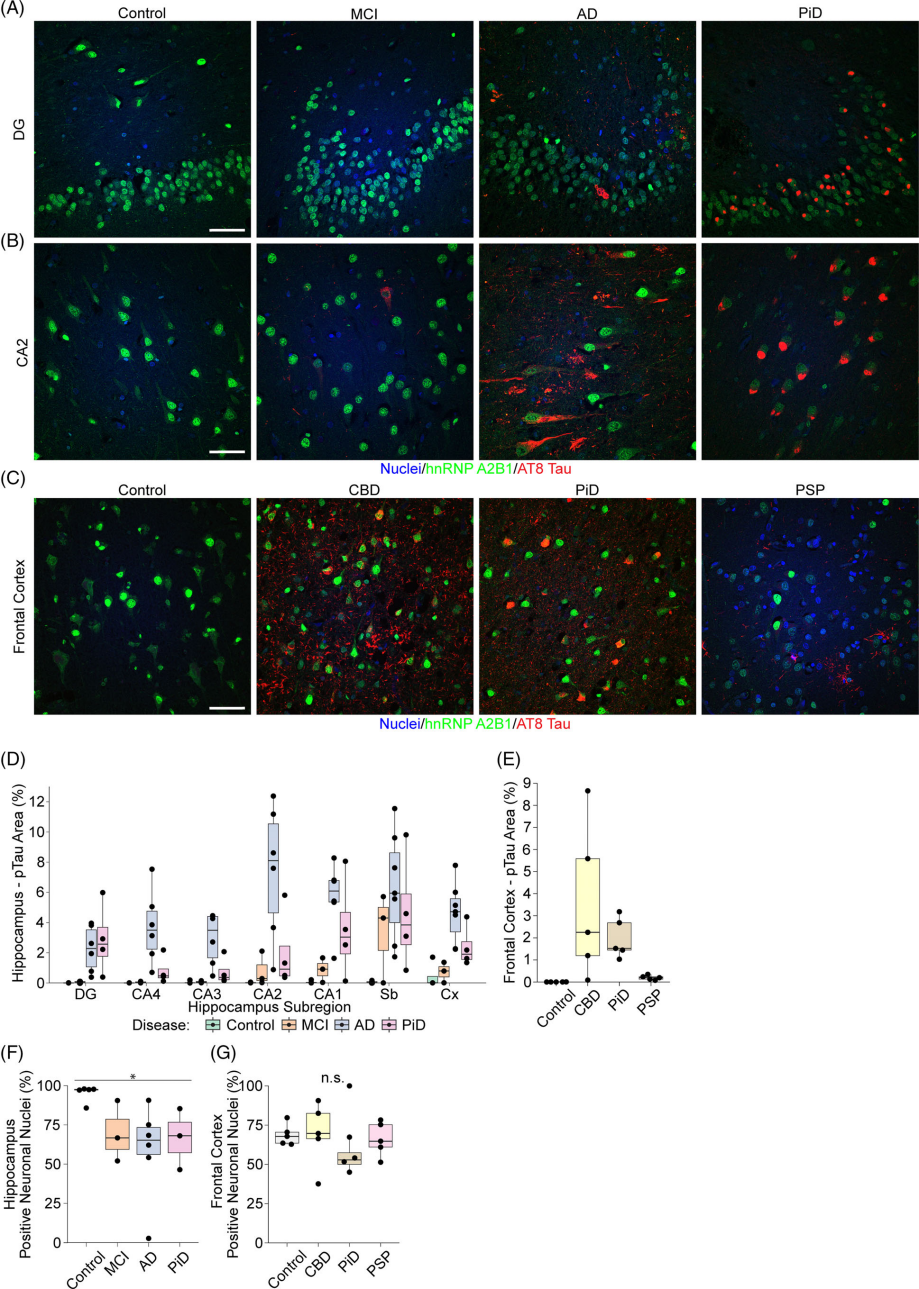
Heterogeneous nuclear ribonucleoprotein (hnRNP) A2B1 expression patterns. Composite micrographs of (A) the DG in the hippocampus from control, mild cognitive impairment (MCI), Alzheimer's disease (AD), and Pick's disease (PiD) cases stained for hnRNP A2B1, phosphorylated tau (pTau) (AT8), and Hoescht (nuclei); (B) micrographs of the CA2 subregion of the hippocampus from control, MCI, AD, and PiD cases stained for hnRNP A2B1, pTau (AT8), and Hoescht (nuclei); (C) micrographs of the frontal cortex from control, corticobasal degeneration (CBD), PiD, and progressive supranuclear palsy (PSP) cases stained for hnRNP A2B1, pTau (AT8) and Hoescht (nuclei). (D) Boxplots showing the percentage load (percentage area with positive staining) of pTau across hippocampal subregions for controls (*n* = 5), MCI (*n* = 3), AD (*n* = 6), and PiD (*n* = 4). (E) Boxplots showing the percentage load (area) of pTau in the frontal cortex of Controls (*n* = 5), CBD (*n* = 5), PiD (*n* = 5) and PSP (*n* = 5). (F) Boxplots showing the percentage of neurons with positive nuclear staining of hnRNP A2B1 in each group (counted manually from the CA2 subregion of the hippocampus, controls *n* = 5, MCI *n* = 3, AD *n* = 6, and PiD *n* = 3). (G) Boxplots showing the percentage of neurons with positive nuclear stain for hnRNP A2B1 in the frontal cortex for each group (Controls *n* = 5, CBD *n* = 5, PiD *n* = 5 and PSP *n* = 5). Scale bar = 50 μM. *Group‐wide significance *p* < 0.05. All images are maximum projections. Boxplots represent the median values, lower and upper quartiles (75% and 25%, respectively) and whiskers represent the quartile to minimum/maximum value but extending no further than 1.5 times the interquartile range. Abbreviations: CA, cornu ammonis, DG, dentrate gyrus; Sb, subiculum; Cx, cortex, n.s., not significant.

In the hippocampus, hnRNP A2B1 was consistently observed as strong nuclear staining in controls (Figure 4A,B). In contrast, MCI and AD cases showed a reduced frequency and intensity of neuronal hnRNP A2B1 staining (Figure 4A,B). PiD cases showed very dull staining compared to other cases but ~70% of neuronal nuclei remained hnRNP A2B1‐positive. Analysis of the number of neuronal nuclei with hnRNP A2B1 staining (irrespective of the intensity of staining) revealed a significant group‐wide reduction in the numbers of hnRNP A2B1 neuronal nuclei in CA2 in neurodegenerative disease in comparison to controls (*p* = 0.033, Kruskal–Wallis test, Figure 4F). In the control frontal cortex, there was a lower proportion of neuronal nuclei stained for hnRNP A2B1 (~75%) in comparison to CA2 (~100%), similar to the trend observed for hnRNP A1 (Figure 4F,G). The proportion of neuronal nuclei stained for hnRNP A2B1 was similar in the frontal cortex from control, CBD, PiD, and PSP cases (Figure 4G); however, it was noted that PSP neurons displayed very wide ranges in hnRNP A2B1 levels with a few very bright cells observed (Figure 4C). This was observed in all 5 PSP cases examined, suggesting that it was a consistent disease feature.

**FIGURE 5:**
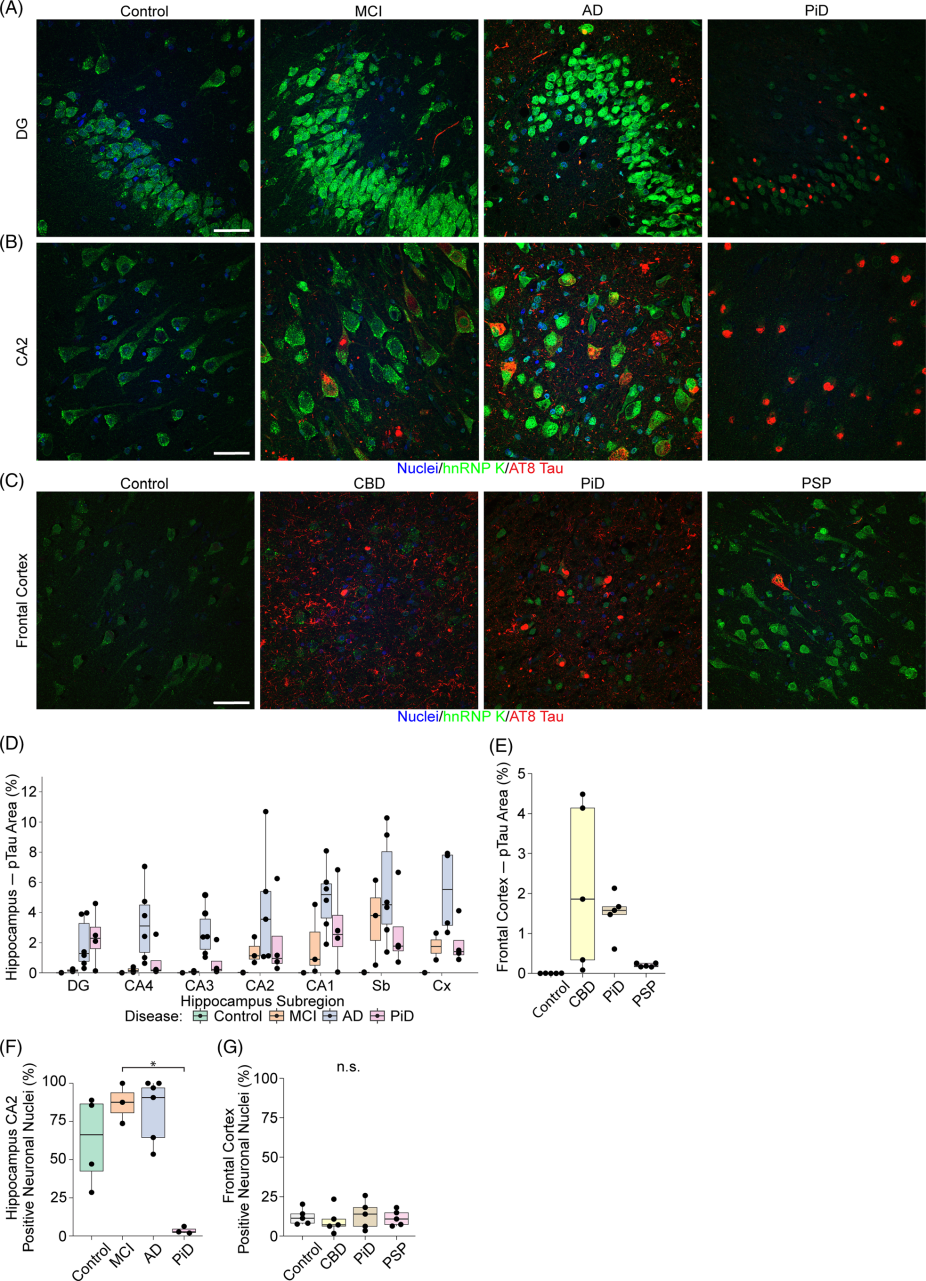
Heterogeneous nuclear ribonucleoprotein (hnRNP) K expression patterns. Composite micrographs of (A) the DG in the hippocampus from control, mild cognitive impairment (MCI), Alzheimer's disease (AD), and Pick's disease (PiD) cases stained for hnRNP K, phosphorylated tau (pTau) (AT8), and Hoescht (nuclei); (B) micrographs of the CA2 subregion of the hippocampus from control, MCI, AD, and PiD cases stained for hnRNP K, pTau (AT8), and Hoescht (nuclei); (C) micrographs of the frontal cortex from control, corticobasal degeneration (CBD), PiD, and progressive supranuclear palsy (PSP) cases stained for hnRNP K, pTau (AT8), and Hoescht (nuclei). (D) Boxplots showing the percentage load (percentage area with positive staining) of pTau across hippocampal subregions for controls (*n* = 4), MCI (*n* = 3), AD (*n* = 6), and PiD (*n* = 4). (E) Boxplots showing the percentage load (area) of pTau in the frontal cortex of Controls (*n* = 5), CBD (*n* = 5), PiD (*n* = 5), and PSP (*n* = 5). (F) Boxplots showing the percentage of neurons with positive nuclear staining of hnRNP K in each group (counted manually from the CA2 subregion of the hippocampus, controls *n* = 4, MCI *n* = 3, AD *n* = 6, and PiD *n* = 3). (G) Boxplots showing the percentage of neurons with positive nuclear stain for hnRNP K in the frontal cortex for each group (Controls *n* = 5, CBD *n* = 5, PiD *n* = 5, and PSP *n* = 5). Scale bar = 50 μM. All images are maximum projections. *p < 0.05. Abbreviations: CA, cornu ammonis; DG, dentate gyrus, Sb, subiculum; Cx, cortex; n.s., not significant.

hnRNP K showed a consistently higher level of mislocalization than hnRNP A1 and hnRNP A2B1 in all cases studied (including controls) in both the hippocampus and frontal cortex. In controls, hnRNP K staining was predominantly in the neuronal cytoplasm, with comparatively less hnRNP K present in the nuclei. Cytoplasmic puncta were often observed in controls and were a particularly predominant feature of MCI (Figure 5A,B). hnRNP K staining in AD typically had a different morphology of strong staining in the cytoplasm and often the nucleus, which was more consistently distributed rather than punctate (Figure 5A,B). In contrast, staining was almost completely depleted in PiD in both the DG and CA2 (Figure 5A,B). Counts of the number of neuronal nuclei that were hnRNP K‐positive confirmed this qualitative observation: hnRNP K‐stained neuronal nuclei were significantly decreased in PiD CA2 in comparison to MCI (Figure 5F). hnRNP K staining in the frontal cortex had a different profile: minimal hnRNP K staining was observed in controls, CBD and PiD, while greater staining was observed in PSP that was predominantly cytoplasmic (Figure 5C). Despite these qualitative differences, counts of the number of hnRNP K‐positive neuronal nuclei (irrespective of staining intensity) showed similarly low levels of hnRNP K neuronal staining in controls, CBD, PiD, and PSP (Figure 5G).

### 3.3 hnRNP expression is complex and varies across subregions

**FIGURE 6:**
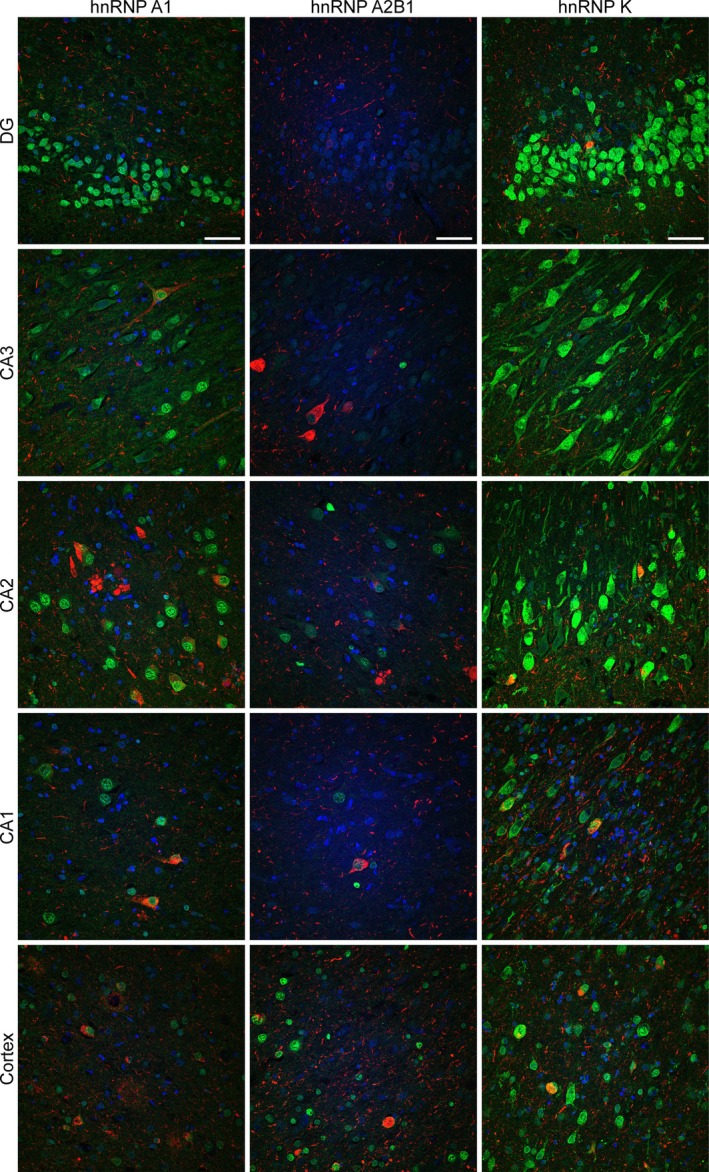
Montage of heterogeneous nuclear ribonucleoproteins (hnRNPs) and AT8 expression across hippocampal subregions. Micrographs of hnRNP A1, hnRNP A2B1 and hnRNP K from the DG, CA3, CA2, CA1, and cortical subregions of the hippocampus. Blue = Hoescht (nuclei), green = hnRNP, red = phosphorylated tau (pTau) (AT8). Scale bar = 50 μM. All images are maximum projections. Abbreviations: CA, cornu ammonis; DG, dentate gyrus.

Our mislocalization analysis showed that the expression patterns of hnRNP A1, hnRNP A2B1, and hnRNP K all vary widely by disease and subregion. Figure 6 shows an example of the complexity and variation in staining patterns that can be seen just in a single section from one AD case. In this example, hnRNP A1 and hnRNP K are strongly expressed in DG, CA3, and CA2. hnRNP A1 staining is noticeably lower in the temporal cortex, whereas hnRNP K staining remains comparable in the temporal cortex to that in the hippocampus. In this case, hnRNP A1 staining is predominantly nuclear, whereas hnRNP K staining is predominantly cytoplasmic, often present in a punctate manner, with frequent strong nuclear staining also observed in most regions except for CA1. In contrast, hnRNP A2B1 staining appears depleted in all regions, with the most prominent staining observed being infrequent nuclear staining in the temporal cortex (Figure 6). These staining patterns also varied by case, therefore preventing the identification of a unified disease phenotype in the small cohort of cases included in this study.

### 3.4 hnRNPs do not strongly colocalise with MC1, AT270, or AT180

To further characterize the colocalization of hnRNPs with pathological tau we performed additional fluorescent immunostains for each hnRNP and the tau markers MC1 (misfolded tau), AT270 (pT181), and AT180 (pT231). We observed minimal colocalization with any of these tau epitopes and hnRNP A1. In the hippocampus, we observed only a few instances of small hnRNP A1 puncta in neuropil threads positive for AT270 as well as one instance of AT270 that may be within the nucleus of a neuron (Figure S5). Similarly, the frontal cortex had minimal colocalization between hnRNP A1 and MC1, AT270, or AT180. We did observe occasional colocalization between small hnRNP A1 puncta and MC1‐, AT270‐, and AT180‐positive puncta (Figure S6). The most frequently observed type of hnRNP A1 dysregulation was reduced expression in disease cases which typically occurred in cells without large tau aggregates.

hnRNP A2B1 also only occasionally colocalized with MC1, AT270 or AT180 tau (Figures S7 and S8). Most tau puncta and aggregates did not colocalize with hnRNP A2B1 (Figures S7 and S8). We observed larger puncta of hnRNP A2B1 in the processes of neurons, but these also appeared free of tau immunoreactivity. The most frequent colocalization occurred between small tau and hnRNP A2B1 puncta (Figures S7 and S8, arrows). hnRNP K followed similar patterns, with minimal colocalization between hnRNP K aggregates and tau (Figures S9 and S10). We only rarely observed colocalization between small hnRNP K puncta (not aggregates) and tau, typically in cell processes and not cell bodies. In sum, colocalization between any hnRNP and tau epitope was uncommon in our cohort.

### 3.5 hnRNP K aggregates are not positive for p62 or G3BP1

**FIGURE 7:**
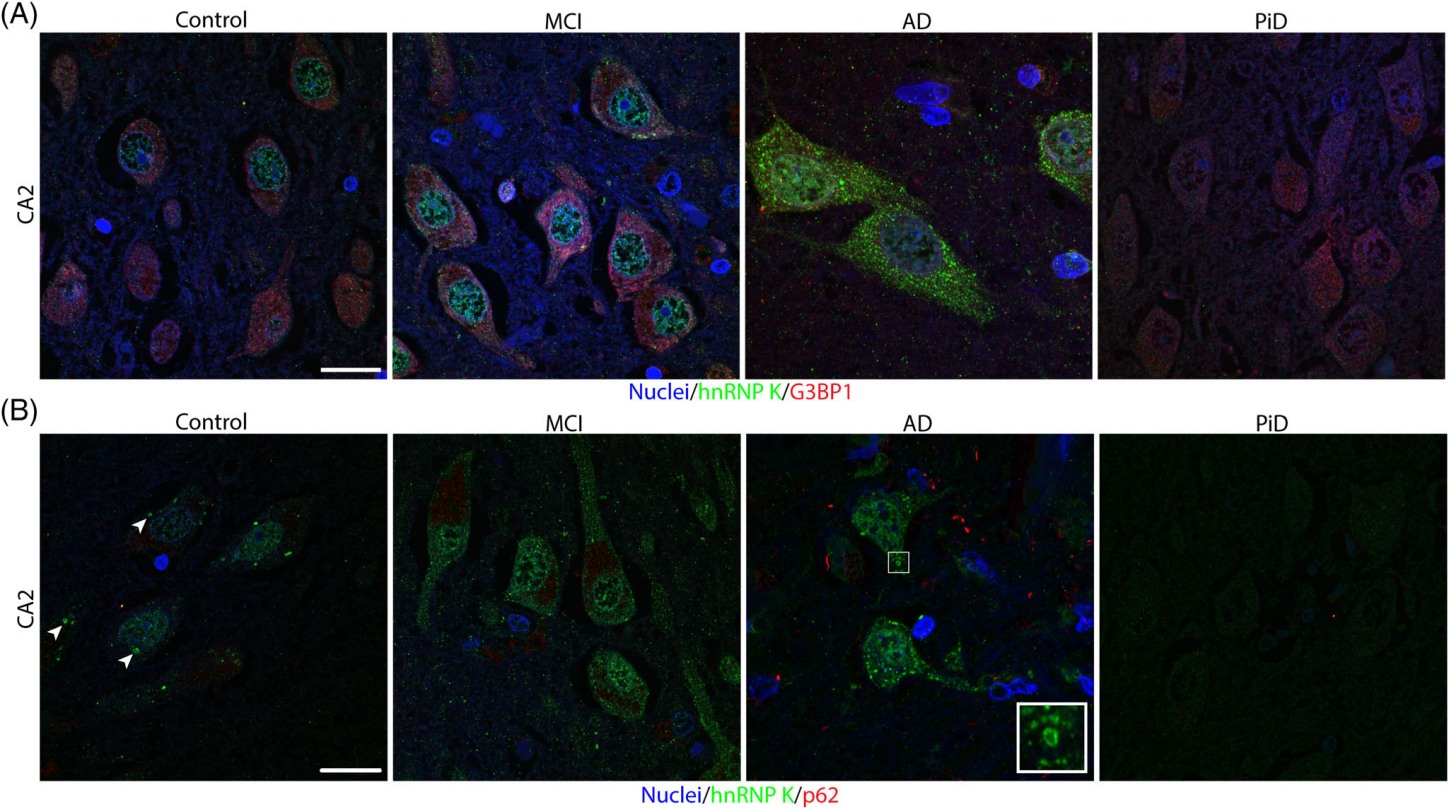
Heterogeneous nuclear ribonucleoprotein (hnRNP) K puncta do not colocalize with p62 or G3BP1. (A) Confocal micrographs of G3BP1 (red) and hnRNP K (green) in the CA2 of the hippocampus. (B) Confocal micrographs of p62 (red) and hnRNP K (green) in the CA2 of the hippocampus. Blue = Hoescht (nuclei). Scale bar = 20 μM. Images are a single slice. Arrowheads indicate hollow hnRNP K puncta. Inset is an example of a large hollow hnRNP K formation (inset size encompassing approximately 4.8 μM^2^). Abbreviations: CA, cornu ammonis; DG, dentate gyrus.

We were interested in determining whether the distinctive hnRNP K‐positive puncta reflected hnRNP K accumulation in stress granules or autophagosomes. Sections were costained with hnRNP K and G3BP1 and p62. G3BP1 presented as expected with diffuse staining observed in most cells and occasional puncta in the cytoplasm. The vast majority of hnRNP K puncta were not G3BP1 positive (Figure 7A) in any disease group, and the rare co‐occurrence of hnRNP K and G3BP1 was observed appeared to be coincidental. p62 staining strongly reflected tau pathology in AD cases, with minimal staining observed in non‐AD cases. While we again observed a rare overlap between p62 staining and hnRNP K puncta (Figure 7B), the majority of hnRNP K puncta were not p62 positive. Interestingly our higher resolution confocal images of hnRNP K puncta collected for this analysis showed that many larger hnRNP K puncta appeared hollow and biased toward the outside of the cell soma (Figure 7A,B).

## 4 DISCUSSION

Our results show that hnRNP A1, hnRNP A2B1, and hnRNP K do not colocalize with AT8‐immunoreactive pTau pathology in human brain sections from cases of AD, MCI, CBD, PiD, or PSP. Nor do they consistently colocalize with MC1 (misfolded tau), AT270 (pT181), or AT180 (pT231) tau in a follow‐up study (Figures [Link], [Link]). We did, however, observe extensive mislocalization of hnRNP A1, hnRNP A2B1, and hnRNP K in our cohort of tauopathy cases. Contrary to our hypothesis, not only did hnRNPs and pTau not colocalize in large tau aggregates but often appeared to be specifically excluded from pTau aggregates. There was some colocalization between small hnRNP puncta and small tau puncta in cell processes, particularly in AD for hnRNP A2B1 and hnRNP K, but this was uncommon and not consistently present in every small tau puncta or neuropil thread. Furthermore, dysregulation of each hnRNP (mislocalization, expression changes, aggregation) were commonly observed in cells without pTau aggregates. While this was surprising in the context of consistent evidence of hnRNP‐tau interactions [1, 2, 3, 5, 6, 8], our results are consistent with a previous proteomics study that found no evidence of enrichment of hnRNP A1, hnRNP A2B1 or hnRNP K in neurofibrillary tangles in comparison to control neurons [35], and previous hnRNP immunohistochemistry studies showing no evidence of a staining pattern resembling tau aggregates in FTLD‐tau cases [11, 15]. Our new results extend these previous studies by confirming the consistent lack of colocalization between four epitopes of pTau and three hnRNPs in five types of tauopathy.

Our results do not directly contradict previous results reporting interactions between hnRNPs and tau in interactome studies. The lack of pTau‐hnRNP colocalization in AT8‐immunoreactive tau aggregates observed in our study does not preclude an interaction between hnRNPs and nonfibrillar species of tau that are not located in large tau aggregates (such as oligomers or monomers). Convincing evidence of an interaction between oligomeric tau constructs and hnRNP A1, hnRNP A2B1, and hnRNP K was recently reported, with hnRNP A2B1 identified the principal oligomeric tau interactor of all proteins assessed [5], highlighting the important role of hnRNPs in disease. The hnRNP mislocalization that we, and others [5, 11, 36], have observed could be an important indicator of the dysregulated and disease‐associated activities of hnRNPs in tauopathies. For example, mislocalization or dysregulation of essential RNA binding proteins (such as hnRNPs) would result in a significant alteration of function and could be the reason for splicing defects seen in tauopathies [11, 13, 19]. Future studies directly comparing the interaction between hnRNPs with monomeric tau and oligomeric tau would be essential to clarify if hnRNPs interact specifically with tau oligomers in comparison to monomeric tau. We frequently observed pTau in a perinuclear presentation around neuronal nuclei (e.g., Figure 5A, MCI) and multiple instances of disrupted nuclear membranes (Figure 1B,D, PSP). We only observed one cell that may have genuine signal in the nucleus of a neuron at observable brightness (Figure S5, AT270 in MCI). The presence of nuclear tau is interesting and observed in several other studies [37, 38], but we rarely observed it in our cohort. However, this may be because of relatively lower intensity of pTau in the nucleus compared to the larger pathological aggregates we focused on.

The mislocalization pattern we observed for each hnRNP was complex: There was limited consistency in the type of staining patterns within each disease group and the mislocalization profiles also widely varied within neighboring brain regions and even neighboring cells. There was also limited consistency between the type or extent of mislocalization between each of the hnRNPs, suggesting that the mislocalization of each of these hnRNPs could be unrelated to each other. These findings could reflect the highly regulated and complex functions of each of these hnRNPs [39]. The analysis of all hnRNPs in the same cases provided us with increased context about how the mislocalization of each hnRNP was related to each other. The consistent mislocalization we saw for hnRNP K in controls, which was not seen for hnRNP A1 or hnRNP A2B1, could suggest that hnRNP K mislocalization is an earlier event in aging‐related diseases. This fits with previous findings suggesting that the mislocalization of hnRNP K into cytoplasmic puncta significantly increases with age but worsens in disease [11], which would explain the presence of these puncta in our aged cohort. While it is still unknown if mislocalization of hnRNP A1 or hnRNP A2B1 is also age‐affected, the staining present in our control cases was more reflective of the reported physiological profile of nuclear‐predominant staining [36, 40, 41]. The high magnification images of hnRNP K revealed a large hollow morphology typically close to the surface of the cell soma. These puncta were negative for p62 and G3BP1 indicating they were not stalled autophagosomes or stress granules. An alternative possibility is that these large hollow hnRNP K deposits may be dysregulated mitochondria. Previous studies have reported colocalization of hnRNP K with peroxiredoxin 5 (PRDX5) to sequester hnRNP K during oxidative stress and regulate osteoclast differentiation and have similarities to these puncta [42].

The dysregulation of hnRNPs also extended to expression in possible non‐neuronal cell types. Most of the alternative cell types expressing hnRNP A2B1 appear to be oligodendrocytes in the frontal cortex, although there remains a population of cells positive for hnRNP A2B1 that did not costain for any of the cell markers used here. In the hippocampus, we observed both microglia and oligodendrocytes expressing hnRNP K. Microglia appeared to have large hnRNP K puncta throughout the cell body and had processes surrounding large hnRNP K aggregates. Oligodendrocytes appeared to have a mix of nuclear and perinuclear signal for hnRNP K. There was also a number of cells not positive for any of these markers nor obviously of the same morphology that were positive for hnRNP K. The alterations in cell type expression of hnRNPs, particularly hnRNP A2B1 and hnRNP K, are interesting and may warrant further exploration to determine how these hnRNPs alter the dynamics of the glial cells. This may be especially pertinent for microglia which appear to be taking up aggregates of hnRNP K.

The complexity of hnRNP mislocalization highlights an important limitation for our current study and key considerations for future studies: a larger sample size is required for future studies to definitively map mislocalization patterns of hnRNPs in tauopathies and across brain regions. While our study was able to provide new descriptive observations about hnRNP mislocalization in tauopathies, it was too underpowered to statistically compare mislocalization between disease groups. The stark difference in mislocalization pattern that we observed between neighboring brain regions highlights an important consideration for future studies in that changes in one brain region may not reflect changes in other brain regions. Utilization of serial sections or multiplexed staining techniques will be useful in superimposing hnRNP staining to provide context about which specific populations are experiencing dysregulation.

In conclusion, while we have shown that hnRNP A1, hnRNP A2B1, and hnRNP K do not colocalize with AT8‐immunoreactive tau aggregates in AD, MCI, CBD, PiD, and PSP, we report extensive mislocalization of all hnRNPs examined in tauopathies. Mislocalization patterns of hnRNPs were complex and appeared to be brain region‐ and cell‐specific. Future studies are required to accurately define disease‐specific mislocalization patterns. Together, our results support an important role of hnRNPs in tauopathies, which should be more closely examined in future studies.

## AUTHOR CONTRIBUTIONS

E.D. and G.L. conceived the study. T.K., K.B., D.R., E.D. performed the experiments. T.K. was responsible for analysis and figure generation. T.K. and E.D. were responsible for interpretation. G.H. and T.W. provided neuropathological assessment of cases and expert advice about the interpretation of the data. E.D. and T.K. wrote the manuscript with input from coauthors. All authors read and approved the final manuscript.

## FUNDING INFORMATION

This study was supported by funding from Bluesand Foundation to E.D., TDM Foundation to E.D., Alzheimer's Association (AARG‐21‐852072) to E.D., Faculty of Medicine and Health, University of Sydney (FMH EMCR Emerging Star Grant) to E.D., NIH (P01AG060882 and P30AG066512) to T.W. G.M.H. is supported by an NHMRC Senior Leadership Fellowship (1176607). Open access publishing facilitated by The University of Sydney, as part of the Wiley–The University of Sydney agreement via the Council of Australian University Librarians. WOA Institution: The University of Sydney. Consortia Name: CAUL 2023.

## CONFLICT OF INTEREST STATEMENT

The authors declare no conflicts of interest.

## ETHICS STATEMENT

This research project was approved by the Human Research Ethics Committee of the University of Sydney and complies with the statement on human experimentation issued by the National Health and Medical Research Council of Australia. Tissues were selected from a neuropathological series collected by the Sydney Brain Bank through regional brain donor programs in Sydney, Australia. The brain donor programs hold approval from the Human Research Ethics Committees of the South Eastern Sydney Area Health Services and comply with the statement on human experimentation issued by the National Health and Medical Research Council of Australia. Additional human brain tissue specimens were acquired under protocols with Institutional Review Board (IRB) approval at NYU Grossman School of Medicine.

## Supporting information


**Figure S1:** Imaging positions and no primary controls. (a) Sections processed in each batch and treated with only secondary antibody as primary antibody controls. (b) Example overviews of tissue sections with approximate example image locations highlighted by red boxes.

**Figure S2:** Covariate analysis. (a) Age distribution of cases in the hippocampus. (b) Age distribution of cases in the frontal cortex. (c) Sex distribution of cases in the hippocampus. (d) Sex distribution of cases in the frontal cortex. (e) Postmortem delays of cases in the frontal cortex.

**Figure S3:** Pearson's correlation from colocalization results. Boxplots representing Pearson's correlation for colocalization between hnRNP and pTau within the pTau‐positive regions of each image for (a) hnRNP A1 in the hippocampus, (b) hnRNP A1 in the frontal cortex, (c) hnRNP A2B1 in the hippocampus, (d) hnRNP A2B1 in the frontal cortex, (e) hnRNP K in the hippocampus, and (f) hnRNP K in the frontal cortex. Plotting the mean Pearsons' *R* ^2^ (linear correlation between pixel intensities in the region of interest) for images from each subregion or group.

**Figure S4:** Cell type markers. Maximum projections of costained sections for (a) frontal cortex hnRNP A2B1 (green) and GFAP (astrocyte marker, red). (b) Frontal cortex hnRNP A2B1 (green) and IBA1 (microglial marker, red). (c) Frontal cortex hnRNP A2B1 (green) and OLIG2 (oligodendrocyte marker, red). (d) Hippocampus hnRNP K (green) and GFAP (astrocyte marker, red). (e) Hippocampus hnRNP K (green) and IBA1 (microglial marker, red). (f) Hippocampus hnRNP K (green) and OLIG2 (oligodendrocyte marker, red). Scale bar = 20 μM. All images are maximum projections. Solid arrows indicate cell type marker expressing the target hnRNP. Hollow arrows indicate cells expressing the hnRNP but negative for the selected cell type marker. For presentation purposes the OLIG2 (red, subparts c and f) signal is altered differently to GFAP and IBA1.

**Figure S5:** Colocalization of hnRNP A1 and tau markers MC1, AT270 and AT180 in AD and MCI in the hippocampus. Maximum projection micrographs of hippocampal sections stained for hnRNP A1 (green) and tau epitopes (red); MC1 (misfolded tau), AT270 (pT181 tau), and AT180 (pT231 tau). Sections include *n* = 1 MCI (top row) and *n* = 1 AD (bottom row). Representative images were collected from the CA2 subregion of the hippocampus. All images are maximum projections. Solid arrows indicate puncta with colocalization. Scale bar = 20 μM.

**Figure S6:** Colocalization of hnRNP A1 and tau markers MC1, AT270 and AT180 in CBD, PiD and PSP in the frontal cortex. Maximum projection micrographs of frontal cortex sections stained for hnRNP A1 (green) and tau epitopes (red); MC1 (misfolded tau), AT270 (pT181 tau) and AT180 (pT231 tau). Sections include *n* = 1 CBD (top row), *n* = 1 PiD (middle row) and *n* = 1 PSP (bottom row). All images are maximum projections. Solid arrows indicate puncta with colocalization. Scale bar = 20 μM.

**Figure S7:** Colocalization of hnRNP A2B1 and tau markers MC1, AT270 and AT180 in AD and MCI in the hippocampus. Maximum projection micrographs of hippocampal sections stained for hnRNP A2B1 (green) and tau epitopes (red); MC1 (misfolded tau), AT270 (pT181 tau), and AT180 (pT231 tau). Sections include *n* = 1 MCI (top row) and *n* = 1 AD (bottom row). Representative images were collected from the CA2 subregion of the hippocampus. All images are maximum projections. Solid arrows indicate larger puncta with colocalization. Hollow arrows indicate hnRNP A2B1 puncta without pTau. Scale bar = 20 μM.

**Figure S8:** Colocalization of hnRNP A2B1 and tau markers MC1, AT270, and AT180 in CBD, PiD and PSP in the frontal cortex. Maximum projection micrographs of frontal cortex sections stained for hnRNP A2B1 (green) and tau epitopes (red); MC1 (misfolded tau), AT270 (pT181 tau), and AT180 (pT231 tau). Sections include *n* = 1 CBD (top row), *n* = 1 PiD (middle row), and *n* = 1 PSP (bottom row). All images are maximum projections. Solid arrows indicate puncta with colocalization. Hollow arrows indicate larger hnRNP A2B1 puncta without pTau. Scale bar = 20 μM.

**Figure S9:** Colocalization of hnRNP K and tau markers MC1, AT270, and AT180 in AD and MCI in the hippocampus. Maximum projection micrographs of hippocampal sections stained for hnRNP K (green) and tau epitopes (red); MC1 (misfolded tau), AT270 (pT181 tau), and AT180 (pT231 tau). Sections include *n* = 1 MCI (top row) and *n* = 1 AD (bottom row). Representative images were collected from the CA2 subregion of the hippocampus. All images are maximum projections. Solid arrows indicate puncta with colocalization. Hollow arrows indicate larger hnRNP K puncta without pTau. Scale bar = 20 μM.

**Figure S10:** Colocalization of hnRNP K and tau markers MC1, AT270, and AT180 in CBD, PiD and PSP in the frontal cortex. Maximum projection micrographs of frontal cortex sections stained for hnRNP K (green) and tau epitopes (red); MC1 (misfolded tau), AT270 (pT181 tau), and AT180 (pT231 tau). Sections include *n* = 1 CBD (top row), *n* = 1 PiD (middle row), and *n* = 1 PSP (bottom row). All images are maximum projections. Solid arrows indicate puncta with colocalization. Hollow arrows indicate larger hnRNP K puncta without pTau. Scale bar = 20 μM.

## Data Availability

The datasets during and/or analysed during the current study available from the corresponding author on reasonable request.
