# Supplementary figures and images for: hnRNP A1, hnRNP A2B1, and hnRNP K are dysregulated in tauopathies, but do not colocalize with tau pathology
Source: Brain Pathol. 2024 Oct 1;35(3):e13305. doi: 10.1111/bpa.13305 (PMC11961206; doi:10.1111/bpa.13305)

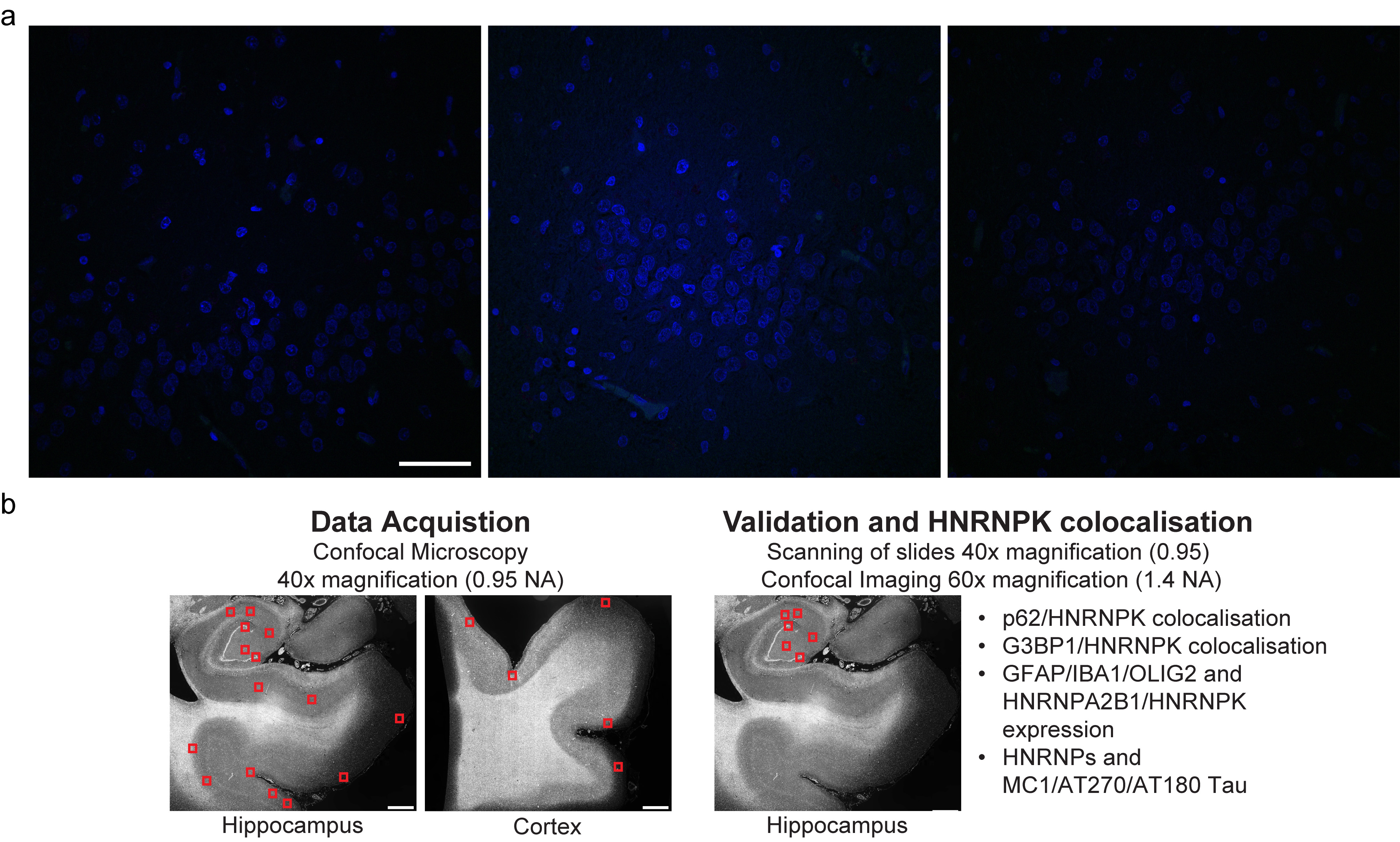

Supplement: Supplementary file 1 — Figure S1: Imaging positions and no primary controls. (a) Sections processed in each batch and treated with only secondary antibody as primary antibody controls. (b) Example overviews of tissue sections with approximate example image locations highlighted by red boxes. [file BPA-35-e13305-s006.jpg]

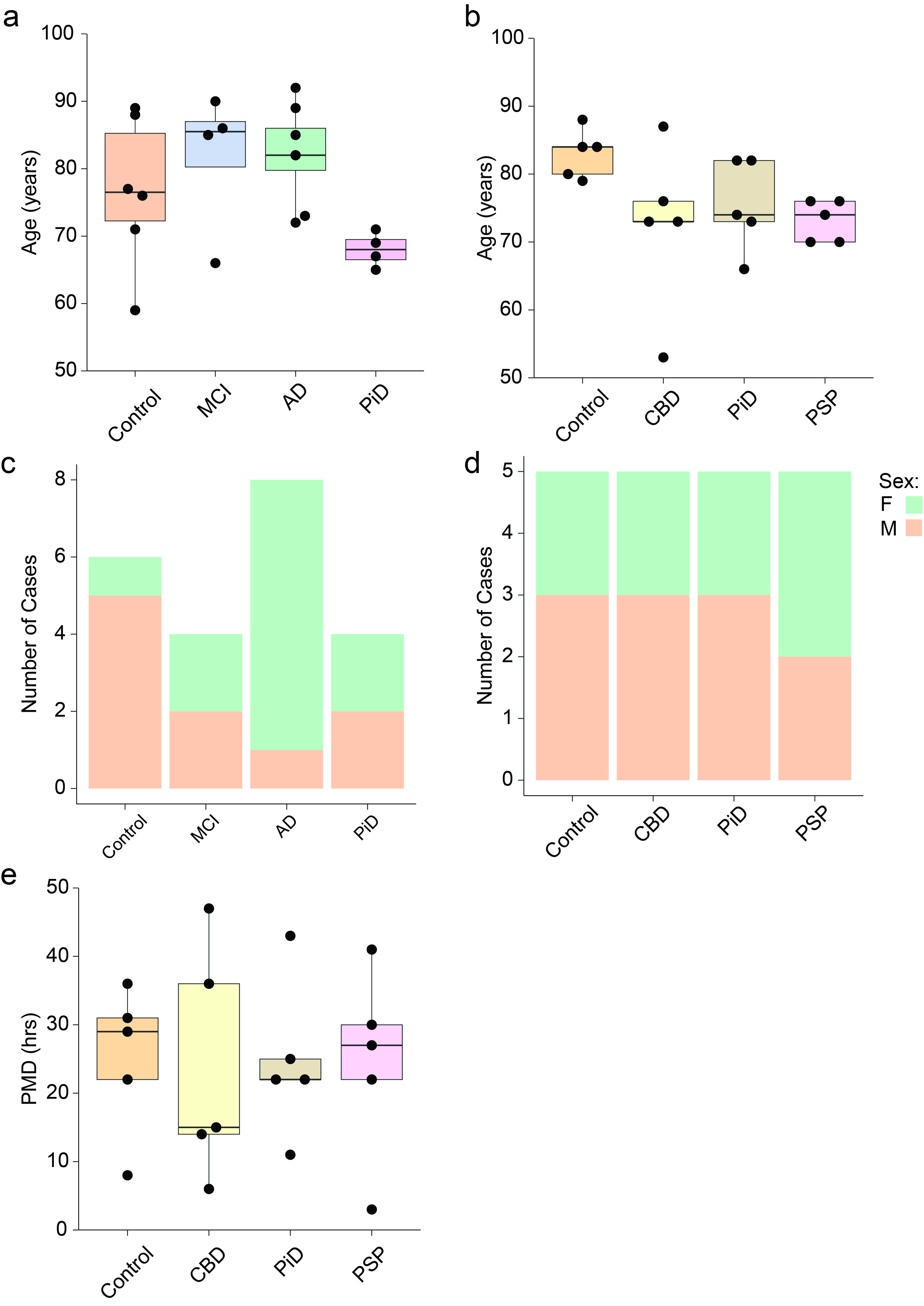

Supplement: Supplementary file 2 — Figure S2: Covariate analysis. (a) Age distribution of cases in the hippocampus. (b) Age distribution of cases in the frontal cortex. (c) Sex distribution of cases in the hippocampus. (d) Sex distribution of cases in the frontal cortex. (e) Postmortem delays of cases in the frontal cortex. [file BPA-35-e13305-s007.tif]

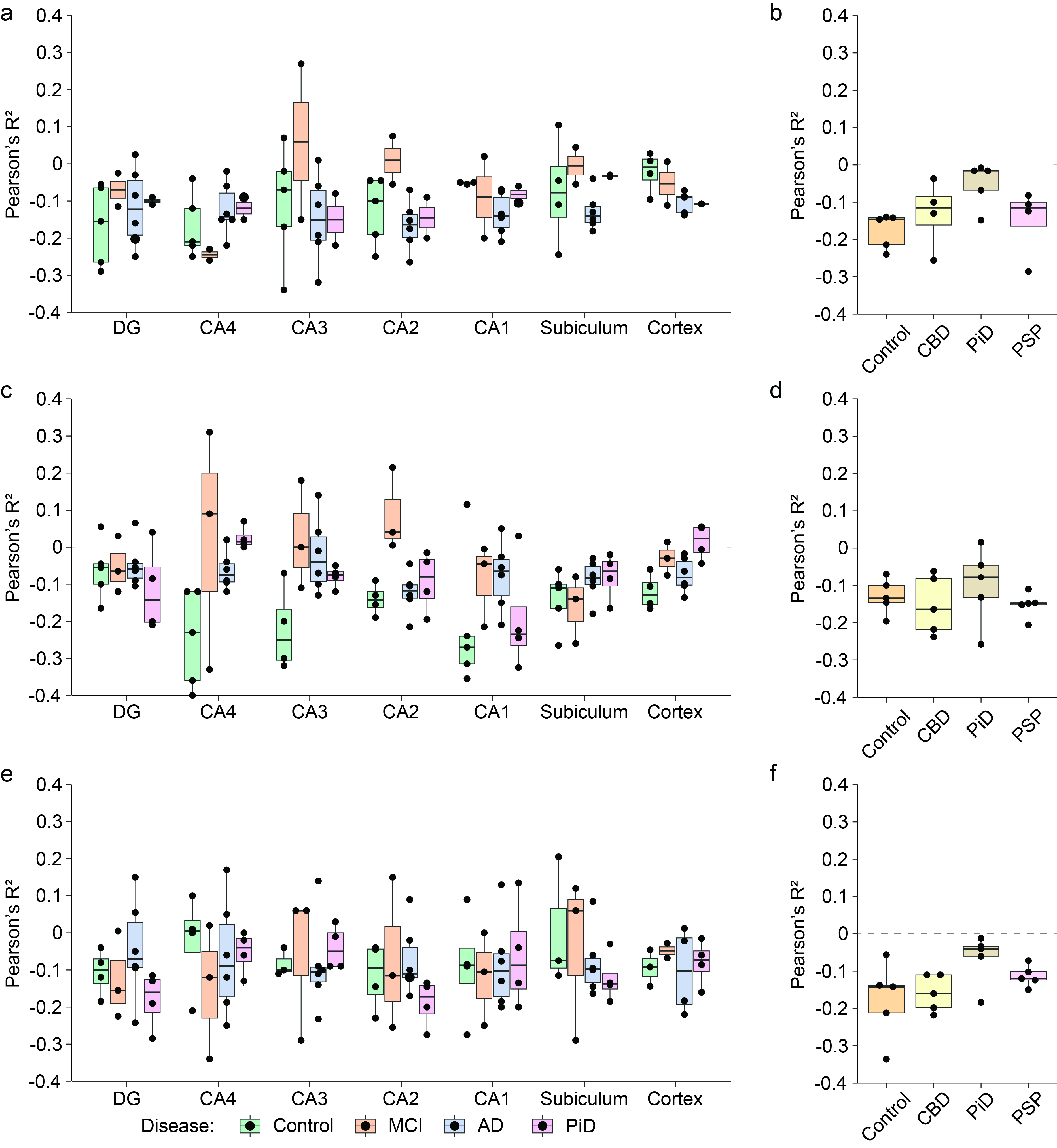

Supplement: Supplementary file 3 — Figure S3: Pearson's correlation from colocalization results. Boxplots representing Pearson's correlation for colocalization between hnRNP and pTau within the pTau‐positive regions of each image for (a) hnRNP A1 in the hippocampus, (b) hnRNP A1 in the frontal cortex, (c) hnRNP A2B1 in the hippocampus, (d) hnRNP A2B1 in the frontal cortex, (e) hnRNP K in the hippocampus, and (f) hnRNP K in the frontal cortex. Plotting the mean Pearsons' R 2 (linear correlation between pixel intensities in the region of interest) for images from each subregion or group. [file BPA-35-e13305-s003.tif]

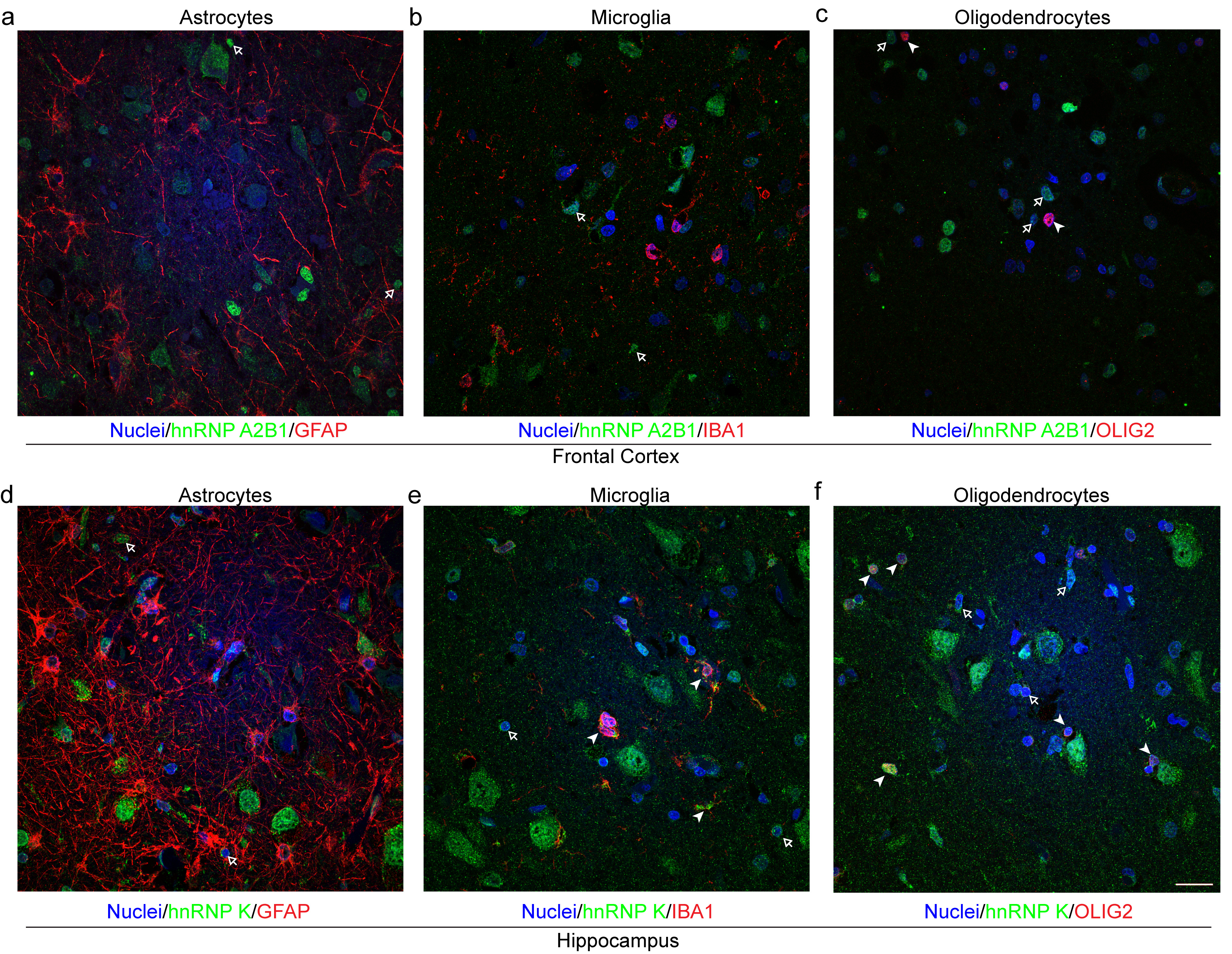

Supplement: Supplementary file 4 — Figure S4: Cell type markers. Maximum projections of costained sections for (a) frontal cortex hnRNP A2B1 (green) and GFAP (astrocyte marker, red). (b) Frontal cortex hnRNP A2B1 (green) and IBA1 (microglial marker, red). (c) Frontal cortex hnRNP A2B1 (green) and OLIG2 (oligodendrocyte marker, red). (d) Hippocampus hnRNP K (green) and GFAP (astrocyte marker, red). (e) Hippocampus hnRNP K (green) and IBA1 (microglial marker, red). (f) Hippocampus hnRNP K (green) and OLIG2 (oligodendrocyte marker, red). Scale bar = 20 μM. All images are maximum projections. Solid arrows indicate cell type marker expressing the target hnRNP. Hollow arrows indicate cells expressing the hnRNP but negative for the selected cell type marker. For presentation purposes the OLIG2 (red, subparts c and f) signal is altered differently to GFAP and IBA1. [file BPA-35-e13305-s010.jpg]

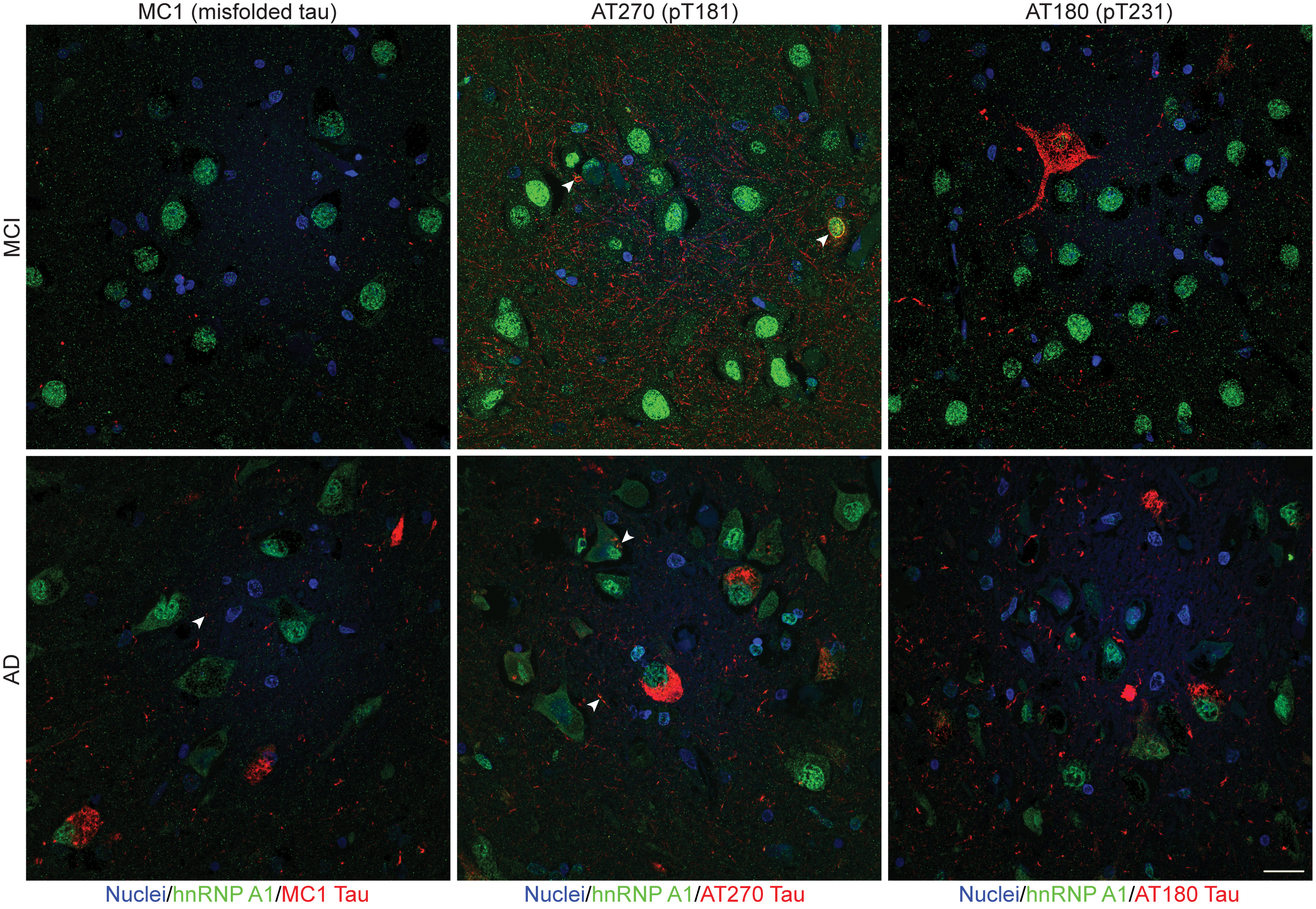

Supplement: Supplementary file 5 — Figure S5: Colocalization of hnRNP A1 and tau markers MC1, AT270 and AT180 in AD and MCI in the hippocampus. Maximum projection micrographs of hippocampal sections stained for hnRNP A1 (green) and tau epitopes (red); MC1 (misfolded tau), AT270 (pT181 tau), and AT180 (pT231 tau). Sections include n = 1 MCI (top row) and n = 1 AD (bottom row). Representative images were collected from the CA2 subregion of the hippocampus. All images are maximum projections. Solid arrows indicate puncta with colocalization. Scale bar = 20 μM. [file BPA-35-e13305-s008.jpg]

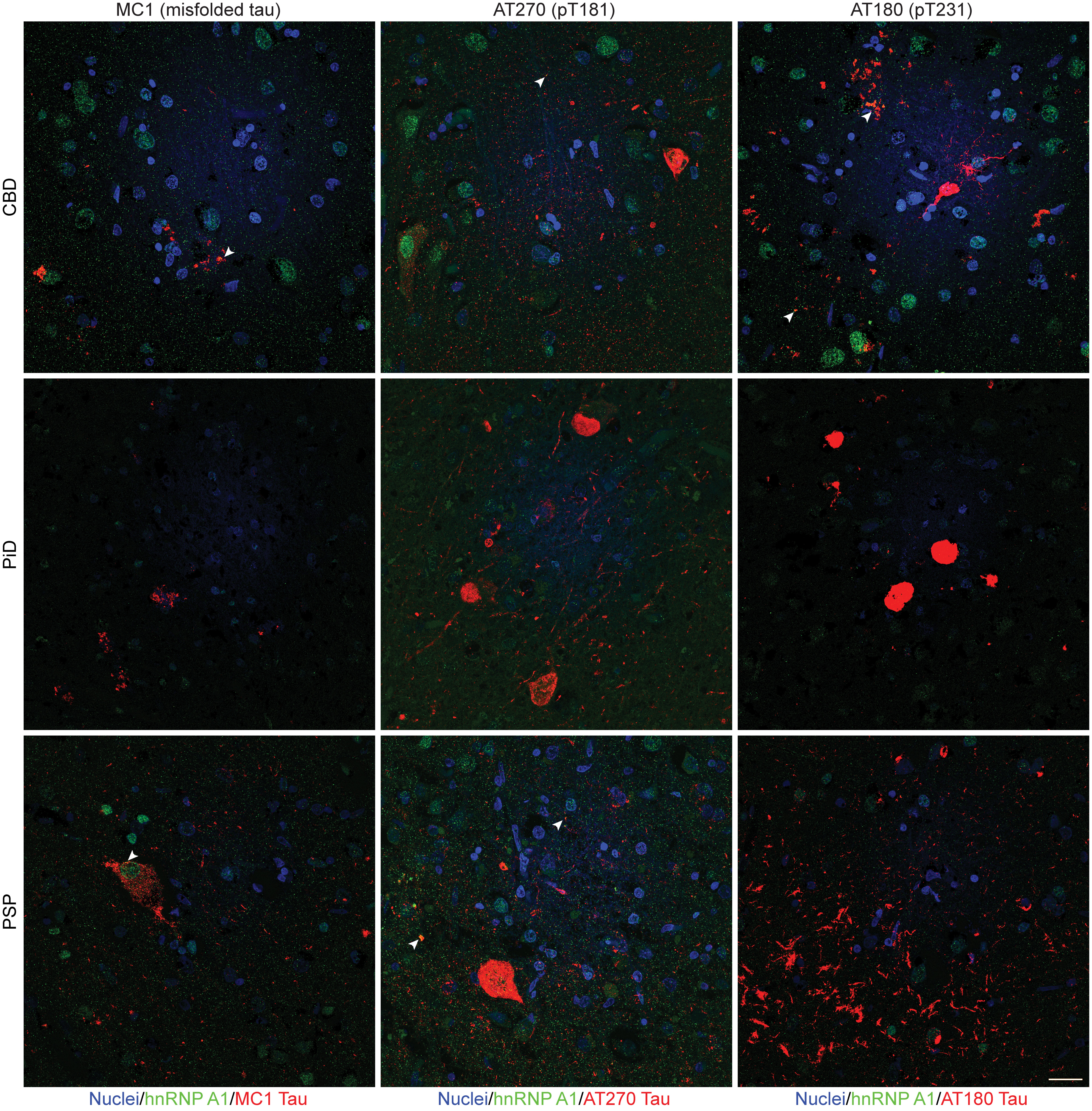

Supplement: Supplementary file 6 — Figure S6: Colocalization of hnRNP A1 and tau markers MC1, AT270 and AT180 in CBD, PiD and PSP in the frontal cortex. Maximum projection micrographs of frontal cortex sections stained for hnRNP A1 (green) and tau epitopes (red); MC1 (misfolded tau), AT270 (pT181 tau) and AT180 (pT231 tau). Sections include n = 1 CBD (top row), n = 1 PiD (middle row) and n = 1 PSP (bottom row). All images are maximum projections. Solid arrows indicate puncta with colocalization. Scale bar = 20 μM. [file BPA-35-e13305-s002.jpg]

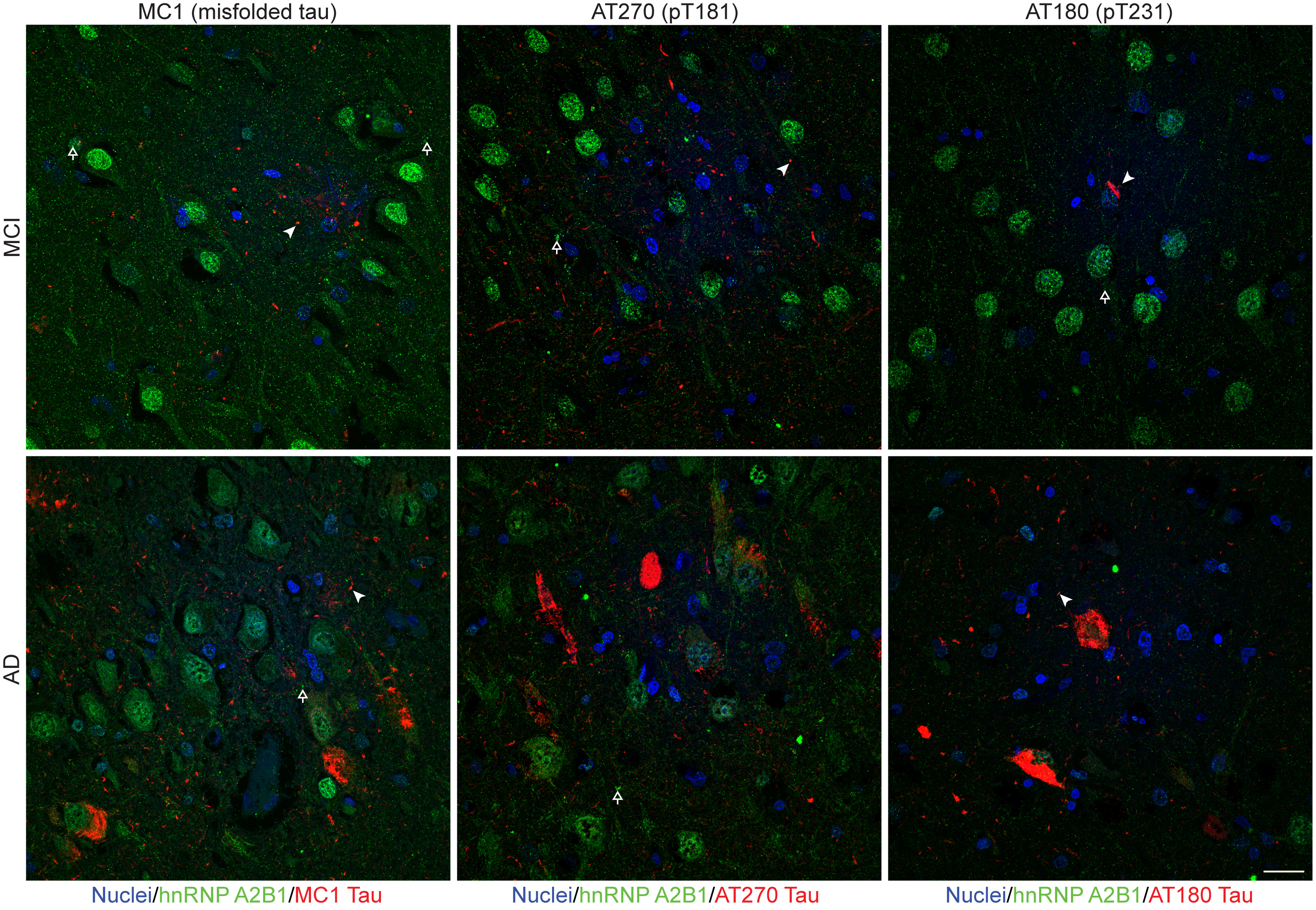

Supplement: Supplementary file 7 — Figure S7: Colocalization of hnRNP A2B1 and tau markers MC1, AT270 and AT180 in AD and MCI in the hippocampus. Maximum projection micrographs of hippocampal sections stained for hnRNP A2B1 (green) and tau epitopes (red); MC1 (misfolded tau), AT270 (pT181 tau), and AT180 (pT231 tau). Sections include n = 1 MCI (top row) and n = 1 AD (bottom row). Representative images were collected from the CA2 subregion of the hippocampus. All images are maximum projections. Solid arrows indicate larger puncta with colocalization. Hollow arrows indicate hnRNP A2B1 puncta without pTau. Scale bar = 20 μM. [file BPA-35-e13305-s004.jpg]

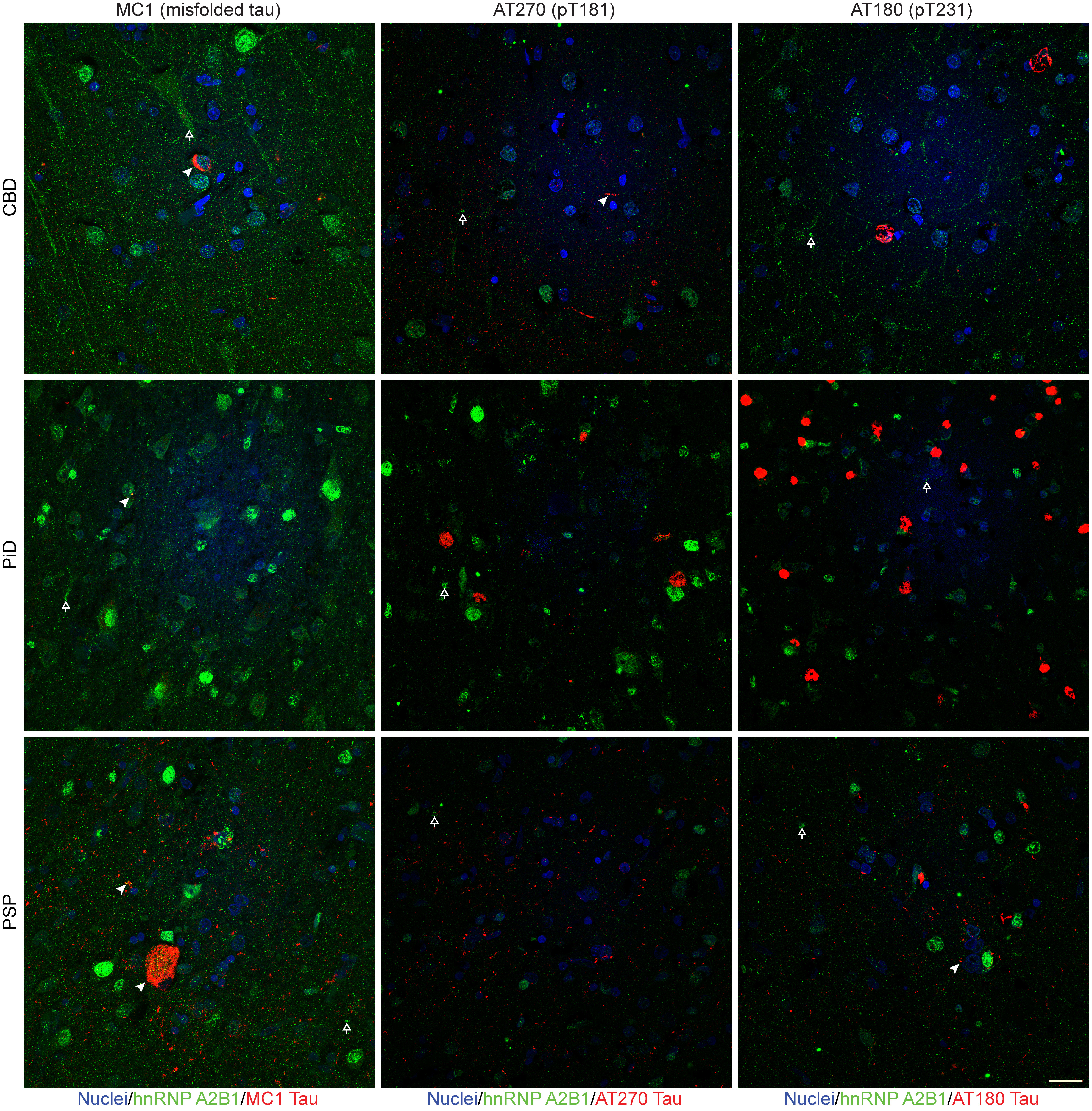

Supplement: Supplementary file 8 — Figure S8: Colocalization of hnRNP A2B1 and tau markers MC1, AT270, and AT180 in CBD, PiD and PSP in the frontal cortex. Maximum projection micrographs of frontal cortex sections stained for hnRNP A2B1 (green) and tau epitopes (red); MC1 (misfolded tau), AT270 (pT181 tau), and AT180 (pT231 tau). Sections include n = 1 CBD (top row), n = 1 PiD (middle row), and n = 1 PSP (bottom row). All images are maximum projections. Solid arrows indicate puncta with colocalization. Hollow arrows indicate larger hnRNP A2B1 puncta without pTau. Scale bar = 20 μM. [file BPA-35-e13305-s001.jpg]

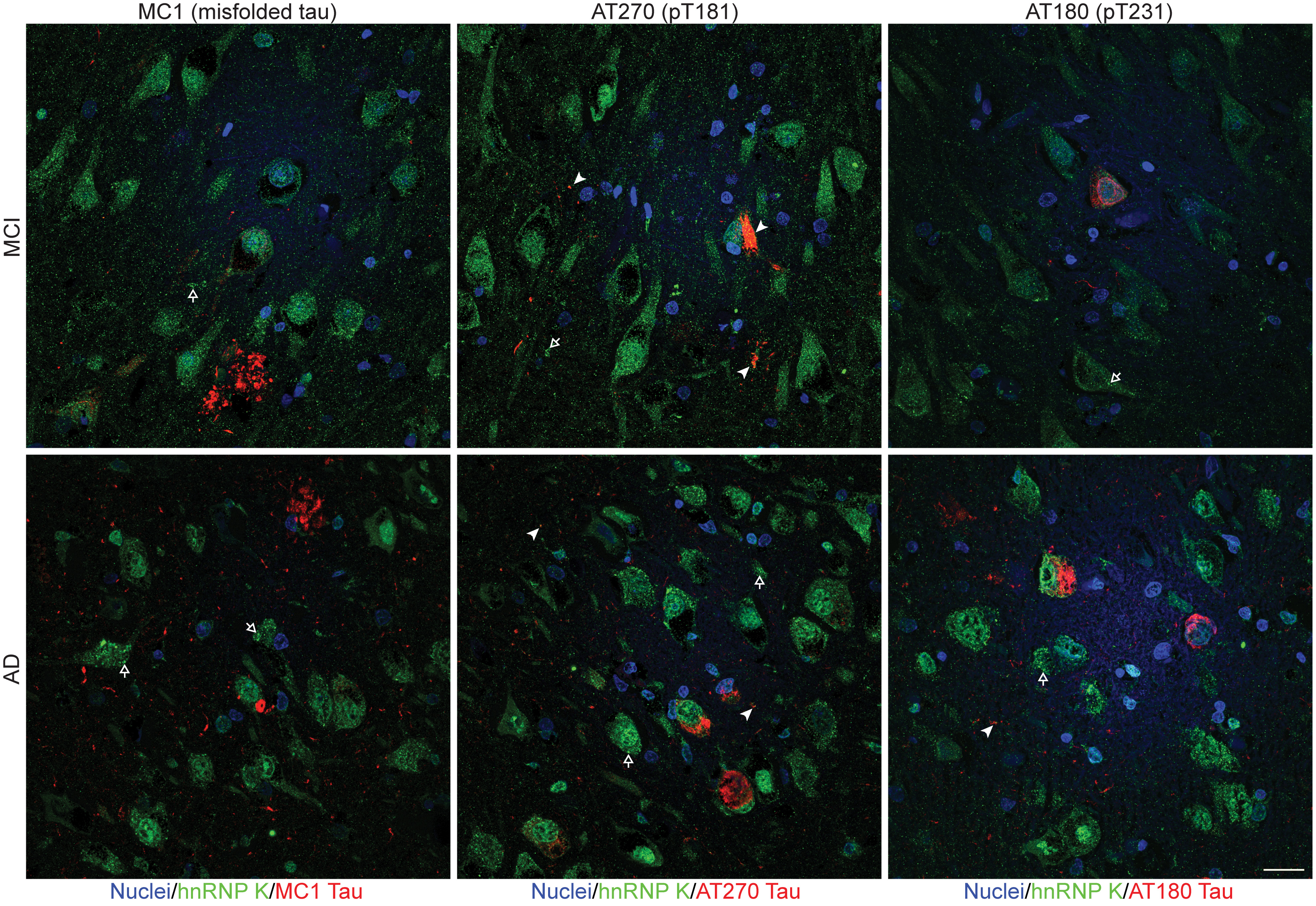

Supplement: Supplementary file 9 — Figure S9: Colocalization of hnRNP K and tau markers MC1, AT270, and AT180 in AD and MCI in the hippocampus. Maximum projection micrographs of hippocampal sections stained for hnRNP K (green) and tau epitopes (red); MC1 (misfolded tau), AT270 (pT181 tau), and AT180 (pT231 tau). Sections include n = 1 MCI (top row) and n = 1 AD (bottom row). Representative images were collected from the CA2 subregion of the hippocampus. All images are maximum projections. Solid arrows indicate puncta with colocalization. Hollow arrows indicate larger hnRNP K puncta without pTau. Scale bar = 20 μM. [file BPA-35-e13305-s009.jpg]

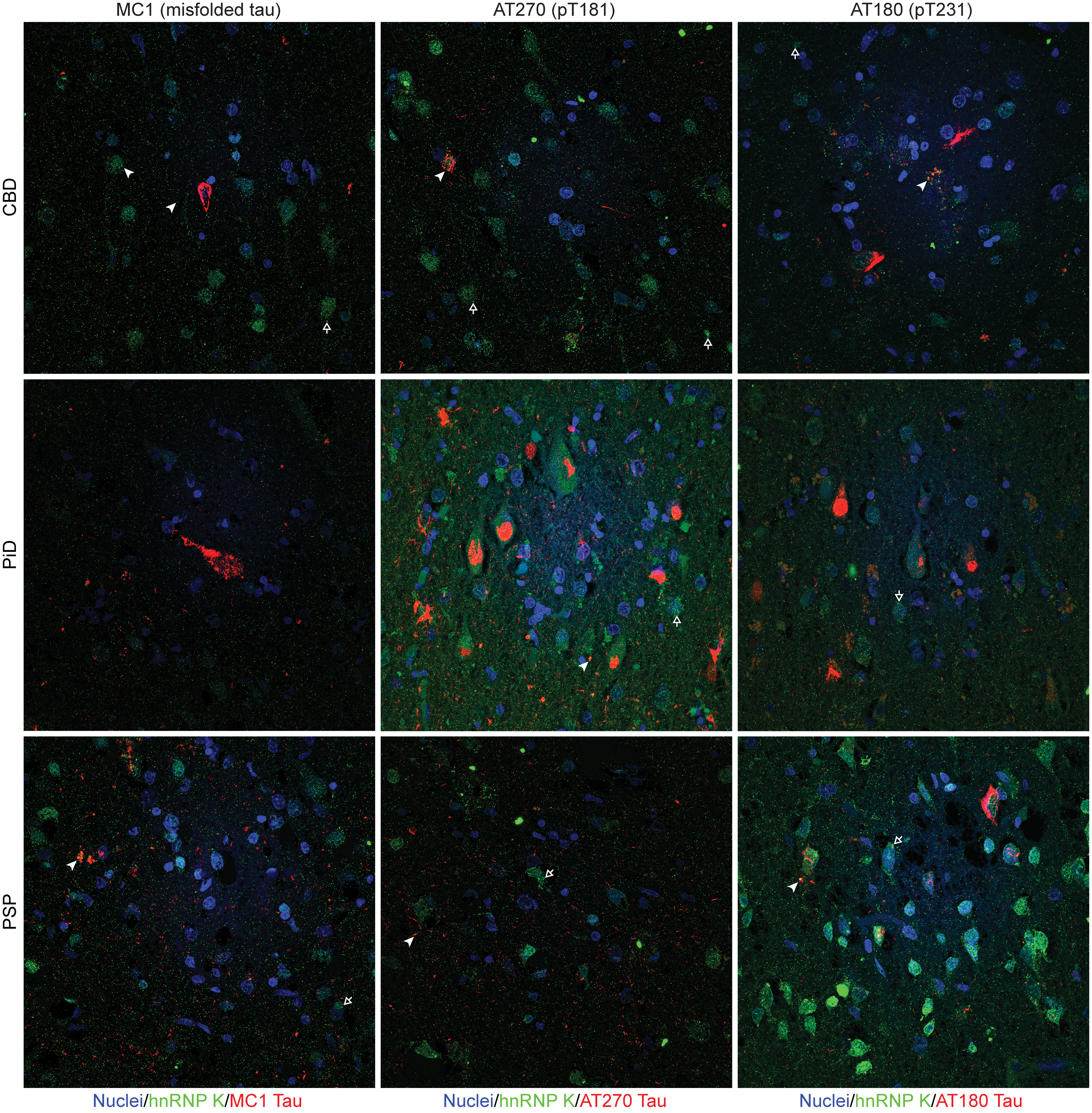

Supplement: Supplementary file 10 — Figure S10: Colocalization of hnRNP K and tau markers MC1, AT270, and AT180 in CBD, PiD and PSP in the frontal cortex. Maximum projection micrographs of frontal cortex sections stained for hnRNP K (green) and tau epitopes (red); MC1 (misfolded tau), AT270 (pT181 tau), and AT180 (pT231 tau). Sections include n = 1 CBD (top row), n = 1 PiD (middle row), and n = 1 PSP (bottom row). All images are maximum projections. Solid arrows indicate puncta with colocalization. Hollow arrows indicate larger hnRNP K puncta without pTau. Scale bar = 20 μM. [file BPA-35-e13305-s005.jpg]
